# B cells shape naive CD8^+^ T cell programming

**DOI:** 10.1172/JCI190106

**Published:** 2025-04-17

**Authors:** Cameron Manes, Miguel Guerrero Moreno, Jennifer Cimons, Marc A. D’Antonio, Tonya M. Brunetti, Michael G. Harbell, Sean Selva, Christopher Mizenko, Tyler L. Borko, Erika L. Lasda, Jay R. Hesselberth, Elena W.Y. Hsieh, Michael R. Verneris, Amanda L. Piquet, Laurent Gapin, Ross M. Kedl, Jared Klarquist

**Affiliations:** 1 Department of Immunology and Microbiology,; 2 Department of Neurology,; 3 Department of Biochemistry and Molecular Genetics,; 4 Department of Pediatrics, Section of Allergy and Immunology, and; 5 Department of Pediatrics, Section of Hematology, Oncology and Bone Marrow Transplantation, University of Colorado School of Medicine, Aurora, Colorado, USA.

**Keywords:** Autoimmunity, Immunology, Vaccines, Adaptive immunity, Immunotherapy, Rheumatology

## Abstract

The presence of B cells is essential for the formation of CD8^+^ T cell memory after infection and vaccination. In this study, we investigated whether B cells influence the programming of naive CD8^+^ T cells prior to their involvement in an immune response. RNA sequencing indicated that B cells are necessary for sustaining the FOXO1-controlled transcriptional program, which is critical for homeostasis of these T cells. Without an appropriate B cell repertoire, mouse naive CD8^+^ T cells exhibit a terminal, effector-skewed phenotype, which significantly impacts their response to vaccination. A similar effector-skewed phenotype with reduced FOXO1 expression was observed in naive CD8^+^ T cells from human patients undergoing B cell–depleting therapies. Furthermore, we show that patients without B cells have a defect in generating long-lived CD8^+^ T cell memory following COVID vaccination. In summary, we demonstrate that B cells promote the quiescence of naive CD8^+^ T cells, poising them to become memory cells upon vaccination.

## Abstract 2

B cells support naïve CD8 T cell homeostasis by sustaining FOXO1, thereby enhancing memory formation, and impacting vaccination success in B cell-depleted patients.

## Introduction

The role of B cells in promoting memory CD8^+^ T cell responses to vaccination, including adjuvanted subunit vaccines and replication-deficient viral vaccine vectors, is well established (1, 2). In B cell–deficient mice, CD8^+^ T cells responding to vaccination show increased effector-skewing and a corresponding decrease in memory-skewing. This leads to a significant reduction in the persistence of antigen-specific cells at memory time points, rendering the mice essentially unprotected against secondary infectious challenge (1). Earlier studies in infection models, such as lymphocytic choriomeningitis virus (LCMV) and *Listeria monocytogenes* (LM), have similarly demonstrated that B cells promote the persistence and protective capacity of CD8^+^ T cell memory (3–6), although the underlying mechanisms were not described.

Many potential mechanisms governing the influence of B cells on vaccine-elicited CD8^+^ T cell responses have been ruled out. Experiments using secreted-IgM-deficient and AID^−/−^ mice demonstrated that B cell secretion of IgM or other immunoglobulin (Ig) isotypes was not involved (1). Although B cells can cross-present antigens in vitro, this did not affect the vaccine response in vivo (1). Importantly, B cells do not indirectly influence the capacity of CD8^+^ T cells to form memory through CD4^+^ T cells. Depletion of CD4^+^ T cells failed to rescue either the peak of the CD8^+^ response or the durability of memory in comparison with similarly treated WT controls (1), nor were CD8^+^ T cell responses to LM altered in CD4^−/−^ mice compared with controls (5).

The situation is further complicated by studies in human populations showing both reduced (2, 7–10) and enhanced (11, 12) CD8^+^ T cell responses in B cell–depleted patients following subunit vaccination. Data from our group (13), as well as from Bar-Or and colleagues (11), identified increased CD8^+^ T cell frequencies in rituximab-treated patients after SARS-CoV-2 mRNA vaccination. This increase was specific to patients with the lowest B cell frequencies and/or vaccine-specific IgG responses. Another study on congenitally B cell–deficient patients with X-linked agammaglobulinemia (XLA) reported that XLA individuals displayed highly functional spike-specific T cell responses, although no direct quantitative comparisons with healthy controls were made (14). These varying observations have yet to be reconciled.

Here, we present data that address the mechanisms behind these seemingly contradictory findings. We show an altered programming of naive CD8^+^ T cells in a B cell–deficient environment in both mice and humans. Changes of FOXO1 activity in CD8^+^ T cells disrupted quiescence, and decreased survival biased T cell responses toward terminal differentiation rather than self-renewing memory. Thus, the B cell environment in which naive CD8^+^ T cells develop significantly influences their response to vaccination or infection.

## Results

### B cells promote memory-fated CD8^+^ T cell responses to vaccination and infection.

**Figure 1:**
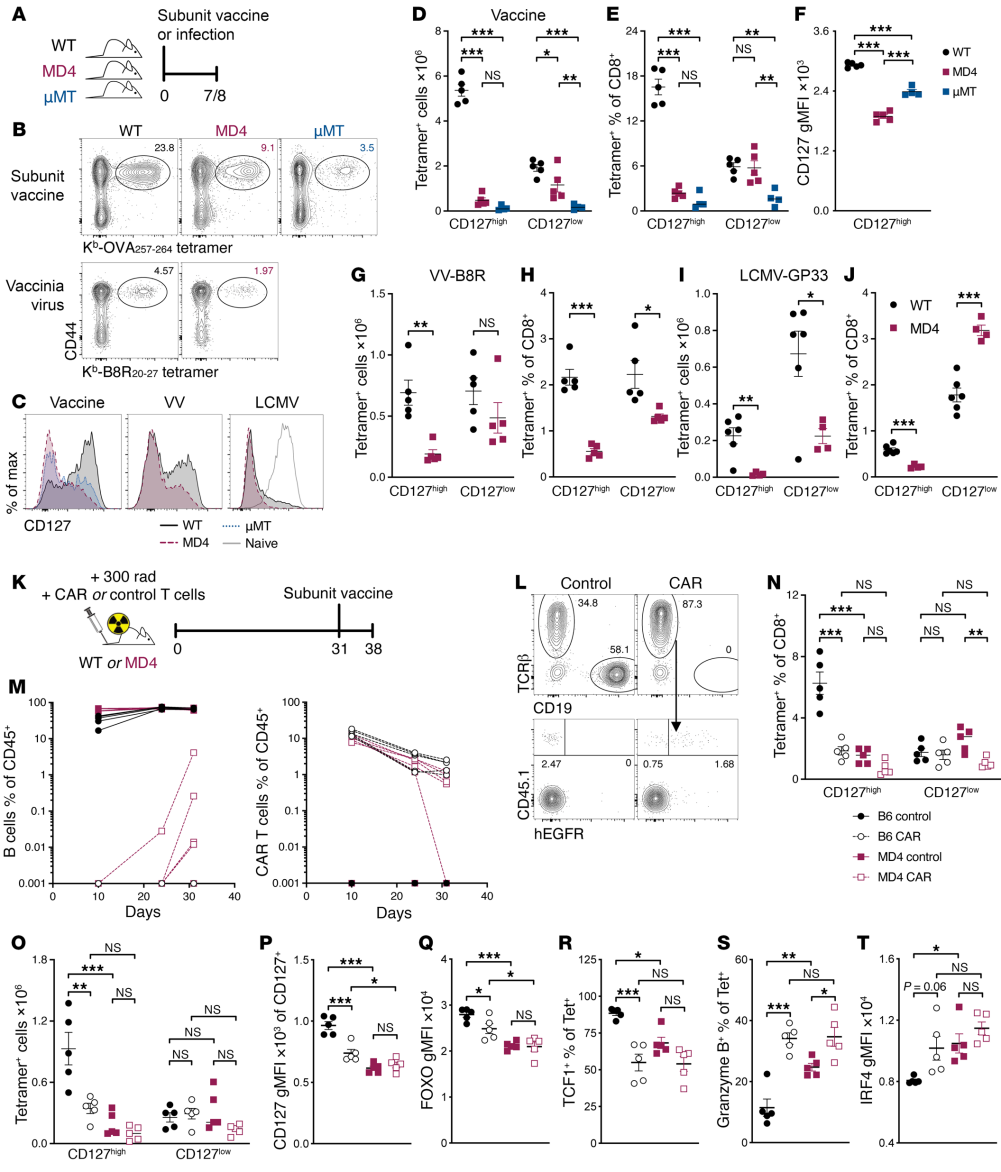
B cells promote memory-fated CD8^+^ T cell responses to vaccination and infection. (**A**–**J**) WT or MD4 mice were either vaccinated with a combined-adjuvant subunit vaccine or infected with vaccinia virus (VV) or LCMV. (**A**) Experimental schematic. (**B**) Representative tetramer staining for pre-gated on live, CD19^−^CD8^+^ lymphocytes. (**C**) Histograms showing CD127 expression by tetramer^+^ cells. Naive (CD44^lo^) CD8^+^ T cells from WT mice provide a high CD127 reference for the LCMV histograms. (**D**–**F**) Seven days after vaccination, spleens were assessed for number (**D**) and percentage (**E**) of SIINFEKL tetramer^+^ cells, and CD127 geometric mean fluorescence intensity (gMFI) for tetramer^+^CD127^hi^ cells (**F**). (**G** and **H**) Seven days after VV infection, spleens were assessed for number (**G**) and percentage (**H**) of B8R tetramer^+^ cells. (**I** and **J**) Eight days after LCMV-Armstrong infection, spleens were assessed for number (**I**) and percentage (**J**) of GP33 tetramer^+^ cells. (**K**–**T**) Sublethally irradiated WT or MD4 mice received 1 million CAR or control T cells. After 30 days, mice were vaccinated with the combined-adjuvant subunit vaccine. (**K**) Experimental schematic. (**L**) PBMCs were assessed for B cells (CD19^+^) and CAR T cells (TCRβ^+^CD45.1^+^hEGFR^+^). (**M**) B cell frequencies (left) and CAR T cell frequencies (right). (**N**–**T**) Seven days after vaccination, spleens were assessed for relative abundance of splenic tetramer^+^ CD127^hi^ cells and CD127^lo^ cells (**N** and **O**); tetramer^+^ cells were assessed for CD127 gMFI (**P**), FOXO1 gMFI (**Q**), percentage positive for TCF1 (**R**), percentage positive for granzyme B (**S**), and IRF4 gMFI (**T**). Data shown are means ± SEM, *n* = 4–5 mice per group, representative of 2 experiments. Significance was defined by 2-way ANOVA (**D**, **E**, and **N**–**T**) or 1-way ANOVA (**F**) with Holm-Šidák multiple-comparison test; *P < 0.05, **P < 0.01, ***P < 0.001.

After a subunit vaccine using a combination adjuvant composed of an agonistic anti-CD40, poly(I:C), and ovalbumin (OVA) (15), we found a paucity of vaccine-elicited CD8^+^ T cells in either μMT^−/−^ mice, which lack B cells, or MD4 mice, a B cell receptor–transgenic (BCR-transgenic) mouse in which more than 90% of the B cells are specific toward hen egg lysozyme, as compared with C57BL/6 (WT) controls (1) (Figure 1, A–E). The primary deficit was in memory-fated CD127^hi^ T cells. Additionally, CD127 (IL-7Rα) expression levels on a per-cell basis were also reduced on these cells (Figure 1F). These phenotypic alterations are functionally relevant, as memory T cells in these B cell–deficient/restricted hosts are defective in their persistence to memory time points and in their capacity for host protection (1), consistent with previously published data on CD8^+^ T cell memory after infection of B cell–deficient mice with LCMV (3, 4), intranasal vaccinia virus (VV) (16), or LM (5). We obtained similar results after infecting WT and MD4 mice with VV or LCMV Armstrong. A reduction in the magnitude of the primary CD8^+^ T cell responses in MD4 mice was evident, particularly for memory-fated cells (Figure 1, A–C and G–J). Infection-elicited CD127^hi^ T cells also showed reduced CD127 expression, while granzyme B^+^ cells exhibited higher granzyme B levels, indicating a more terminally differentiated effector cell response (Supplemental Figure 1, A–H; supplemental material available online with this article; https://doi.org/10.1172/JCI190106DS1). Thus, a B cell–replete environment facilitates T cell memory formation following either vaccination or infection.

To determine whether our results were restricted to genetic models congenitally lacking B cells or with BCR restriction, and to use a more clinically relevant model, we depleted WT mice of B cells using CD19-reactive chimeric antigen receptor (CAR) T cells. Mice were sublethally irradiated, a standard measure to improve the efficiency of CAR T cell grafting (17), before transfer of control or CAR T cells (Figure 1K). Mice were vaccinated 30 days later with poly(I:C), anti-CD40, and OVA (combined-adjuvant protein subunit vaccine). Peripheral blood mononuclear cells (PBMCs) were assessed for the relative abundance of CAR T cells and B cells over time, and all but one mouse maintained B cell levels at or below 0.5% of CD45^+^ lymphocytes, with most below the limit of detection (Figure 1, L and M). This depletion of B cells prior to vaccination led to a remarkable recapitulation of the phenomenon we observed in B cell–deficient and MD4 mice, resulting in significantly fewer memory-phenotype cells, but equivalent numbers of effector cells (Figure 1, N and O). The T cell phenotype in B cell–depleted mice was characterized by relatively low expression of proteins associated with memory, including CD127 (18), FOXO1 (19–23), and TCF1 (24–26), and high expression of markers associated with effector-phenotype cells, including granzyme B (25, 27) and IRF4 (28) (Figure 1, P–T). Notably, depleting B cells in MD4 mice did not rescue the CD8^+^ T cell phenotype; rather, the magnitude of the response was reduced and the responding cells were slightly more terminally differentiated in comparison with control-treated MD4 mice. With consistent results across multiple, distinct models in the context of infection and vaccination, we conclude that the T cell phenotypes observed in MD4 and μMT^−/−^ mice are not due to any confounding factor in those genetic models of congenital B cell dysfunction/deficiency, but rather are due to their lacking some WT B cell function(s) responsible for promoting a memory phenotype.

### B cells shape naive CD8^+^ T cell programming, promoting FOXO1-mediated homeostasis in mice and humans.

**Figure 2:**
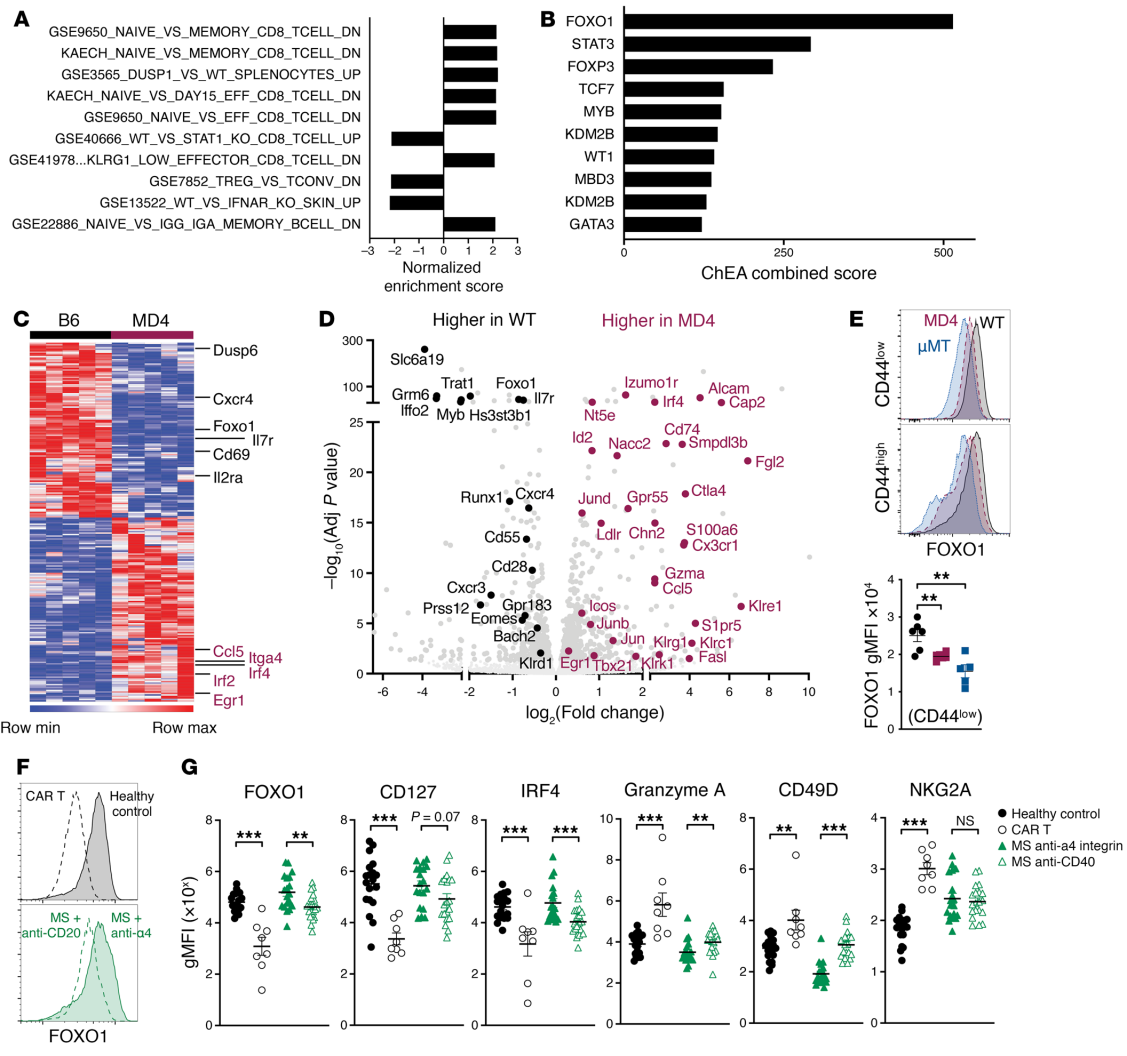
B cells shape naive CD8^+^ T cell programming, promoting FOXO1-mediated homeostasis in mice and humans. (**A**–**D**) Bulk RNA sequencing on naive CD8^+^ T cells (sorted on live CD8^+^CD19^−^B220^−^CD44^lo^) from 5 WT and 5 MD4 mice. (**A**) The top 10 most significant gene sets identified by GSEA using the Broad Institute’s Molecular Signatures Database (MSigDB) immunologic signature gene sets (C7) ordered by adjusted *P* value. (**B**) ChEA analysis of the 1,027 differentially expressed genes by DESeq2 ranked by combined score. (**C**) Heatmap of the 242 genes identified by ChEA as being associated with FOXO1 transcriptional activity. (**D**) Volcano plot where genes known to be differentially expressed in *Foxo1*-deficient cells (31, 33) are highlighted in black (higher in WT) or maroon (higher in MD4). (**E**) Flow cytometry staining of CD8^+^ T cells from WT, μMT^−/−^, and MD4 mice. Representative histograms are shown for CD44^lo^ naive cells (top) and for CD44^hi^ memory and virtual memory cells (middle). A graph plotting the individual gMFI from 5 mice per group for CD44^lo^ naive cells is shown at bottom. Data shown are means ± SEM, representative of more than 2 experiments. (**F**) Representative FOXO1 staining in naive CD8^+^ T cells from healthy control versus CAR T cell–treated patients (top) and patients with MS on anti–α_4_ integrin versus anti-CD20 therapy (bottom). (**G**) Summarized results showing the gMFI values (1 × 10^3^, 10^3^, 10^3^, 10^2^, 10^3^, and 10^2^, respectively) for FOXO1, CD127, IRF4, GZMA, CD49d, and NKG2A protein staining for all 4 groups; *n* = 19, 8, 20, and 19, respectively; **P < 0.01, ***P < 0.001.

Given the influence of B cells on the development of effector and memory CD8^+^ T cells, we set out to determine whether B cells impact the programming of naive CD8^+^ T cells. Bulk RNA sequencing was performed on purified, CD44^lo^ CD8^+^ T cells from unmanipulated WT and MD4 mice, identifying 1,027 differentially expressed genes (DEGs) between the 2 strains. Gene sets identified by gene set enrichment analysis (GSEA) (29) (Figure 2A) or Gene Ontology pathway analysis (Supplemental Figure 2A) showed that these DEGs were largely associated with T cell signaling, activation, and differentiation. ChIP Enrichment Analysis (ChEA) (30), in which transcription factor regulation is inferred by integration of public genome-wide chromatin immunoprecipitation (ChIP) experiments, revealed that nearly one-quarter of these genes (242/1,027) are putatively regulated by FOXO1 (*P* < 1 × 10^−53^ with an odds ratio of 4.0; Figure 2, B and C). These DEGs overlap substantially with those identified in previous studies as both positively and negatively regulated by FOXO1 activity, which antagonizes effector differentiation, promotes memory development (19–22), and constrains activation to maintain the naive state of CD8^+^ T cells (31–33) (Figure 2D). Many of the genes expressed at higher levels in T cells from MD4 mice are linked to activation and differentiation, including *GzmA*, *Fasl*, *Tbx21* (encoding T-bet), *Klrc1* (NKG2A), *Klrk1* (NKG2D), *Klrg1*, *Cx3cr1*, *Irf4*, and *Itag4* (CD49d). Conversely, genes expressed more highly in cells from WT mice included those associated with or involved in maintaining quiescence, like *Foxo1* itself, *Il7r*, *Bach2*, and *Myb*. The diminished FOXO1 activity in MD4-derived naive CD8^+^ T cells implicated by RNA sequencing was confirmed by flow cytometric analysis, with reductions noted in FOXO1 (Figure 2E) and 2 genes it positively regulates, CD127 (34) and eomesodermin (35) (Supplemental Figure 2C). Increased CD122 expression was also observed, which is similarly increased in naive CD8^+^ T cells from *Foxo1^−/−^* mice (32). Moreover, a transition away from quiescence toward differentiation was confirmed, by increases in CD49d, an integrin upregulated in terminal effector cells, and in IRF4, which binds the FOXO1 gene locus (36) and thereby represses memory development (28). Elevated phospho-Akt, p70-S6K, and 4E-BP1 were also noted (Supplemental Figure 2C), indicating increased activation through PI3K and mTOR. These phenotypic changes were confirmed in the CAR T cell experiments described above, where the naive CD8^+^ T cells from B cell–depleted mice exhibited slightly lower FOXO1 and CD127 than naive T cells from control mice (Supplemental Figure 2D). Collectively, these data suggest that FOXO1-mediated regulation of T cell homeostasis is dysregulated in mice lacking WT B cells, predisposing T cells toward effector differentiation and away from quiescence and memory T cell differentiation.

We next characterized naive CD8^+^ T cells in peripheral blood from 4 patient cohorts: (a) healthy control subjects, (b) patients who received anti-CD19 CAR T cell therapy (37) for the treatment of B cell malignancies, (c) patients with multiple sclerosis (MS) who received an α_4_ integrin inhibitor (natalizumab), as a control for MS disease per se, and (d) patients with MS who received B cell–depleting antibody (rituximab or ocrelizumab; Supplemental Table 1). To guard against a possible confounding effect of B cell malignancy on naive T cell phenotype in the CAR T cell therapy group, samples were included only for patients who achieved complete remission and were negative for CD19^+^ B cells at the time of blood draw, since peripheral B cells are strongly correlated with disease relapse (38). B cells were essentially undetectable in the blood of the 8 patients who had received anti-CD19 CAR T cell therapy (Supplemental Figure 2, E–G).

We used high-parameter flow cytometry to identify naive CD8^+^ T cells (Supplemental Figure 2E) and probe them for various markers of differentiation based on the genes we identified as differentially expressed between naive CD8^+^ T cells from WT versus MD4 mice. FOXO1 expression was significantly lower in the 2 patient cohorts depleted of B cells compared with their respective controls (Figure 2, F and G). CD127, which is positively regulated by FOXO1 activity (34), was also lower. In contrast to the mouse data, here we noted significantly lower IRF4 on the CD8^+^ T cells of the B cell–depleted patient populations. Though high IRF4 activity is associated with terminal effector T cell differentiation and can repress FOXO1 gene transcription (28, 36), it is also positively regulated by FOXO1 (39, 40); thus, the lower levels of IRF4 perhaps reflect lower FOXO1 activity. Naive CD8^+^ T cells from the B cell–depleted patient cohorts also expressed higher granzyme A (Figure 2G). Additional markers of differentiation in CD8^+^ T cells corroborated the granzyme A data; CD49d was higher in both B cell–depleted patient populations, and NKG2A was higher in the CAR T cell recipients (Figure 2G). Taken together, these data strongly support a role for B cells in maintaining the quiescent homeostasis of naive human CD8^+^ T cells.

### Naive CD8^+^ T cells from B cell–restricted hosts exhibit normal proliferative capacity, but defective survival.

**Figure 3:**
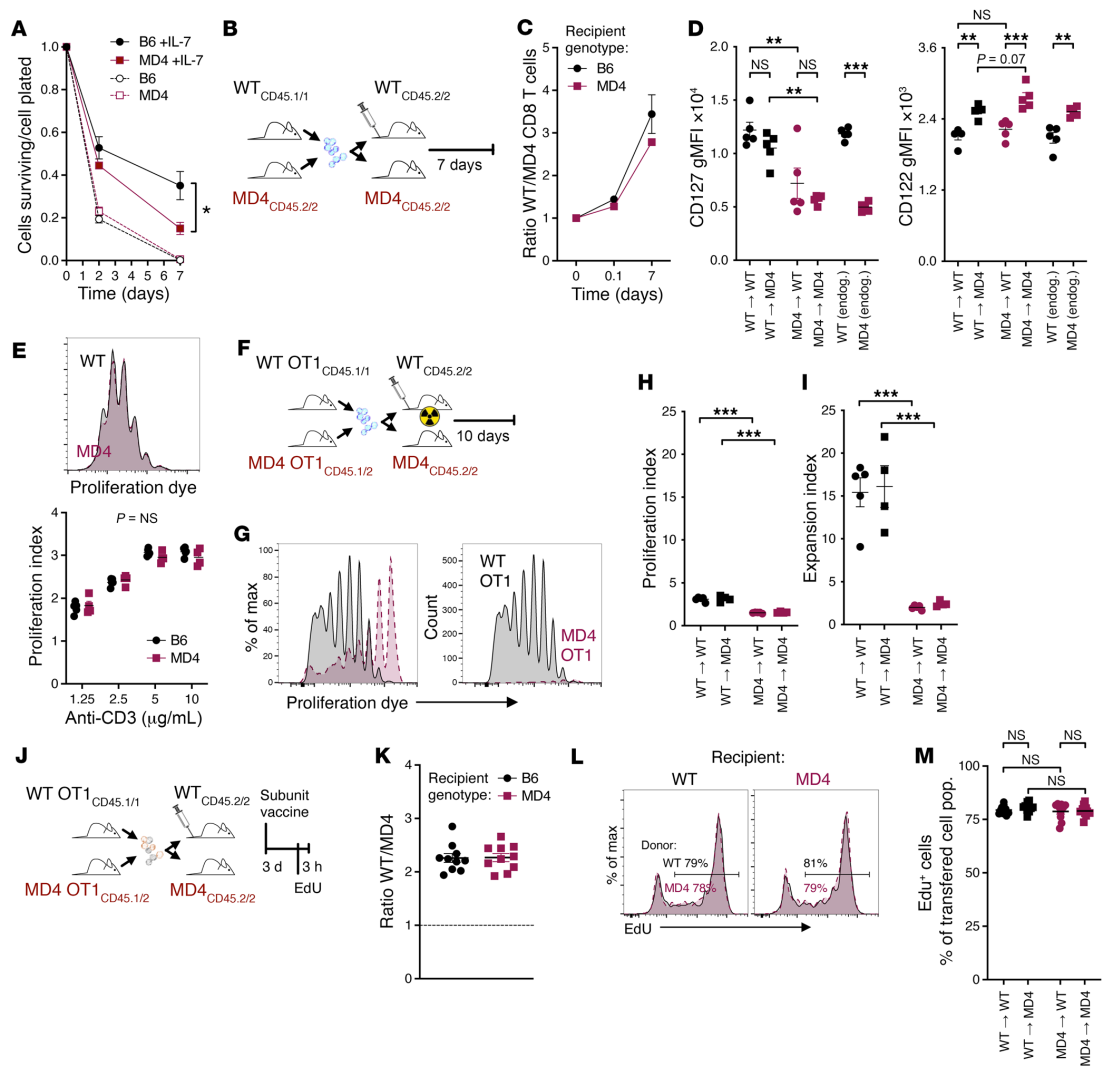
Naive CD8^+^ T cells from B cell–restricted hosts exhibit normal proliferative capacity, but defective survival. (**A**) Purified CD8^+^ T cells were plated with or without 5 ng/mL human IL-7. Ratio of cells surviving to number of cells plated is displayed over time. Data are combined from 3 experiments. Analysis by 2-way ANOVA yielded a significant interaction effect, indicating that the slopes of the two lines differ significantly. (**B**–**D**) Purified CD8^+^ T cells from WT and MD4 mice were dye-labeled, mixed in equal numbers, and cotransferred into WT or MD4 mice. (**B**) Experimental schematic. (**C**) Ratio of transferred cells in blood 2 hours after transfer, and in spleens 7 days later. (**D**) CD127 and CD122 gMFI was analyzed on transferred cells and endogenous naive CD8^+^ T cells on day 7. (**E**) Purified CD8^+^ T cells from WT or MD4 mice were stimulated with plate-bound anti-CD3. Representative proliferation dye dilution (top) and proliferation index (bottom). (**F**–**I**) Purified OT1 T cells from WT or MD4 mice were dye-labeled, mixed in equal numbers, and transferred into sublethally irradiated recipients. Spleens were analyzed 10 days later. (**F**) Experimental schematic. (**G**) Representative proliferation dye dilution as percentage of maximum (left) or as total counts (right). (**H** and **I**) Proliferation (**H**) and expansion (**I**) indices. (**J**–**M**) Purified OT1 T cells from WT and MD4 mice were mixed in equal numbers and transferred to recipient mice, which then received subunit vaccination. (**J**) Experimental schematic. (**K**) Ratio of transferred OT1 T cells at day 3. (**L**) Representative EdU incorporation plots. (**M**) Quantification of EdU incorporation. Data shown are means ± SEM, representative of ≥2 experiments. Significance was defined by 2-way ANOVA with Holm-Šidák multiple-comparison test; *P < 0.05, **P < 0.01, ***P < 0.001.

Naive CD8^+^ T cells are dependent on IL-7 receptor signaling for survival (41, 42). The reduced CD127 (IL-7Rα) expression on naive CD8^+^ T cells from MD4 mice compared with their WT counterparts suggested a potential survival defect. To address this possibility, we purified CD8^+^ T cells from WT or MD4 mice and plated them in vitro with IL-7. Short-term survival was unaffected. However, cells from WT mice survived significantly better than cells from MD4 mice after 7 days in culture (Figure 3A). To test survival in vivo, we cotransferred polyclonal CD8^+^ T cells from WT and MD4 donor mice into WT and MD4 recipient mice (Figure 3B). WT-derived CD8^+^ T cells exhibited superior persistence compared with MD4-derived CD8^+^ T cells, irrespective of the host into which they were transferred (Figure 3C). This survival defect was associated with decreased CD127 (IL-7Rα) and elevated CD122 (IL-2/15Rβ) on naive CD8^+^ T cells, where expression of CD127 was largely dependent on donor cell origin, and CD122 expression was primarily influenced by recipient genotype (Figure 3D). As these data indicated that T cells from MD4 mice had a shorter half-life than normal, we investigated whether this corresponded with the relative abundance of CD8^+^ T cell subpopulations in healthy, unmanipulated mice. Indeed, MD4 mice presented with approximately one-third fewer naive CD8^+^ T cells and three-quarters fewer CD62L^hi^ virtual memory (VM) cells (CD44^hi^CD49d^lo^), whereas they had equivalent numbers of central memory, effector memory, and CD62L^lo^ VM cells (Supplemental Figure 3, A and B). Earlier analyses showed that CD4^+^ T cell numbers, including regulatory T cells, were equivalent, as were XCR1^+^ dendritic cell numbers (1). As with the cotransfer experiments, naive CD8^+^ T cells exhibited significantly lower CD127 and slightly higher CD122 (Supplemental Figure 3, C and D). As IL-15 is important for VM survival, we only noted relatively minor decreases in their CD122 expression. The CD62L^hi^ VM cell number deficit in MD4 mice was more likely due to a loss of CD127, which was the most highly expressed and the most severely reduced in the 2 underrepresented CD8^+^ T cell subpopulations.

Next, we dye-labeled CD8^+^ T cells from WT and MD4 mice and evaluated their proliferative capacity in response to antigen stimulation in vitro and in vivo. Whereas purified WT- and MD4-derived CD8^+^ T cells proliferated equivalently in response to plate-bound anti-CD3 (Figure 3E), their response in vivo differed significantly. Dye-labeled cells were cotransferred into sublethally irradiated WT or MD4 recipients (Figure 3F), and their lymphopenia-induced proliferation (43, 44) was evaluated 10 days later. To avoid the hyperproliferative responses driven by commensal microflora (43), we used OT1 cells, which carry the high-affinity TCR specific for the SIINFEKL peptide from OVA. The number of cell divisions undergone by MD4-derived OT1 T cells was only slightly reduced compared with that of WT-derived OT1 T cells, as quantified by proliferation index (Figure 3, G and H). However, we observed significant differences in T cell accumulation, as measured by the expansion index (Figure 3, G–I). The differences in accumulation were exclusive to the genotype of the donor cells.

As an additional assessment of in vivo proliferation, we measured 5-ethynyl-2′-deoxyuridine (EdU) incorporation 3 days after protein subunit vaccination (Figure 3J), a time point when the majority of responding T cells are actively dividing. The ratio of WT- to MD4-derived OT1 cells was roughly 2:1, regardless of the recipient mouse genotype (Figure 3K). Notably, 80% of the transferred T cells were EdU positive across all groups (Figure 3, L and M), indicating that despite comparable proliferation, MD4-derived cells exhibited reduced accumulation. Altogether, these data demonstrate that naive CD8^+^ T cells isolated from a B cell–restricted environment retain normal proliferation capacity, but impaired survival.

### The B cell environment in which a naive CD8^+^ T cell develops has significant consequences for its response to vaccination.

**Figure 4:**
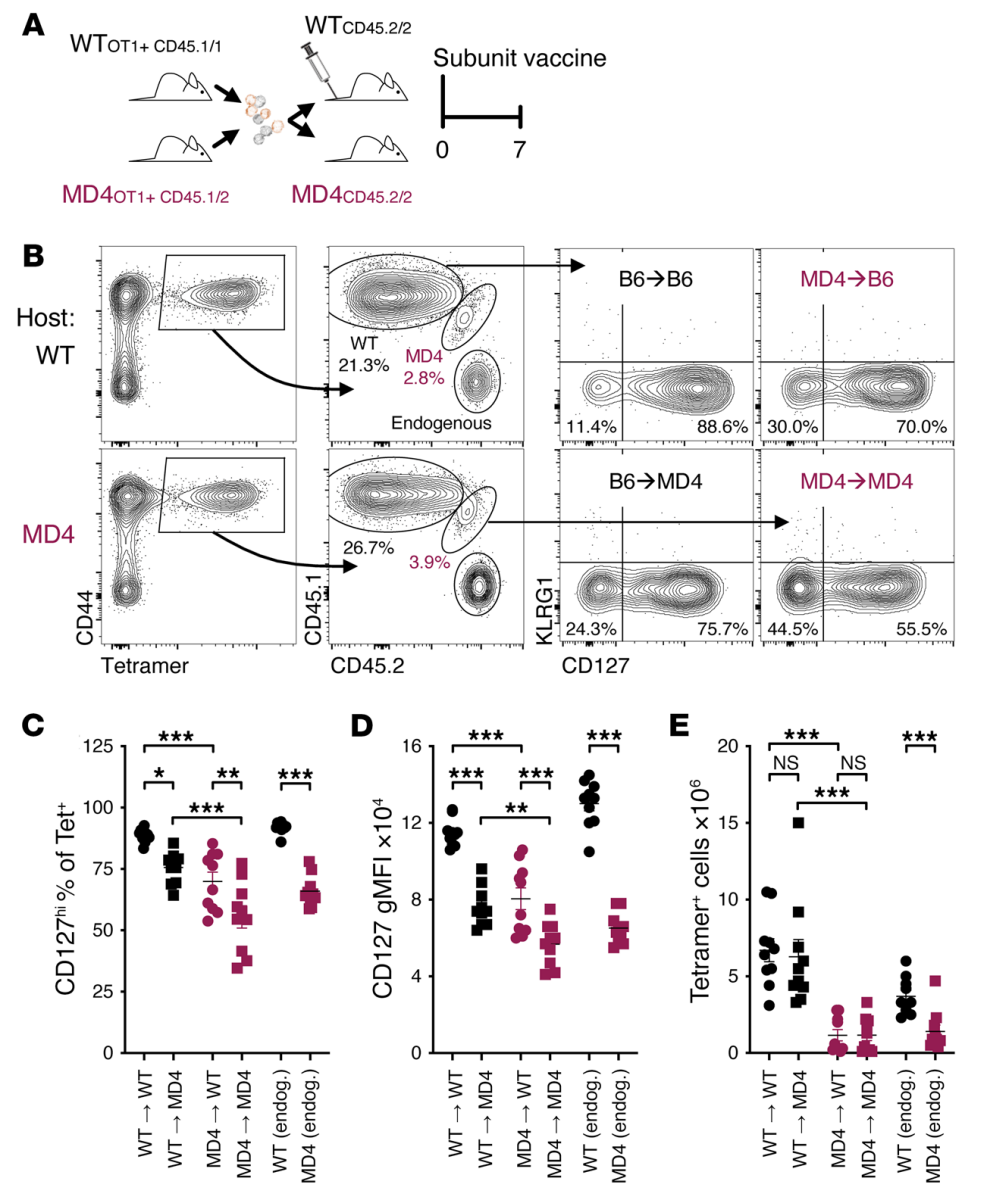
The B cell environment in which a naive CD8^+^ T cell develops has significant consequences for its response to vaccination. Four hundred purified OT1 T cells from WT and MD4 mice were mixed 1:1 and transferred i.v. into WT or MD4 recipients. Mice were then immediately vaccinated with the combined-adjuvant subunit vaccine [OVA, poly(I:C), and anti-CD40]. Seven days later, spleens were analyzed by flow cytometry. (**A**) Experimental schematic. (**B**) Representative tetramer staining on CD19^−^CD8^+^ lymphocytes (left), staining for CD45.1 and CD45.2 (middle) to identify tetramer^+^ cell origin, and staining for CD127 (right). (**C**) Percentage of tetramer^+^ cells positive for CD127. (**D**) The gMFI of CD127 staining on tetramer^+^CD127^hi^ cells. (**E**) Total number of splenic tetramer^+^ cells. Data shown are means ± SEM, *n* = 5 mice per group, representative of 2 experiments. Significance was defined by 2-way ANOVA with Holm-Šidák multiple-comparison test; *P < 0.05, **P < 0.01, ***P < 0.001.

Considering the impact of B cells on naive CD8^+^ T cell cytokine receptor expression and survival, we anticipated that this would exert a dominant influence on the T cell responses to vaccination. OT1 T cells from WT and MD4 mice were cotransferred into recipients of each genotype, followed immediately by combined-adjuvant protein subunit vaccination (Figure 4A). Spleens were harvested 7 days later and assessed for antigen-specific T cells (Figure 4B). Both the recipient and donor genotypes contributed to the phenotype of the response with respect to the percentage that were CD127^hi^ (Figure 4C) and the level of CD127 expressed by CD127^hi^ cells (Figure 4D). The transfer of non-transgenic, polyclonal CD8^+^ T cells yielded similar results (Supplemental Figure 4, A–D). Thus, a B cell–deficient/restricted environment fails to support maximal expression of markers associated with a memory cell fate. In contrast, the overall magnitude of the primary response was dependent on the origin of the donor cells. The number of WT donor cells recovered was more than 5 times that of MD4 donor cells on average, regardless of the recipient mouse genotype (Figure 4E), consistent with the findings from day 3 after vaccination (Figure 3K). Hence, the regulation of naive CD8^+^ T cell programming by B cells greatly influences the ability of CD8^+^ T cells to respond effectively to vaccination.

### B cells limit effector CD8^+^ T cell expansion following mRNA lipid nanoparticle vaccination, preserving memory pool.

**Figure 5:**
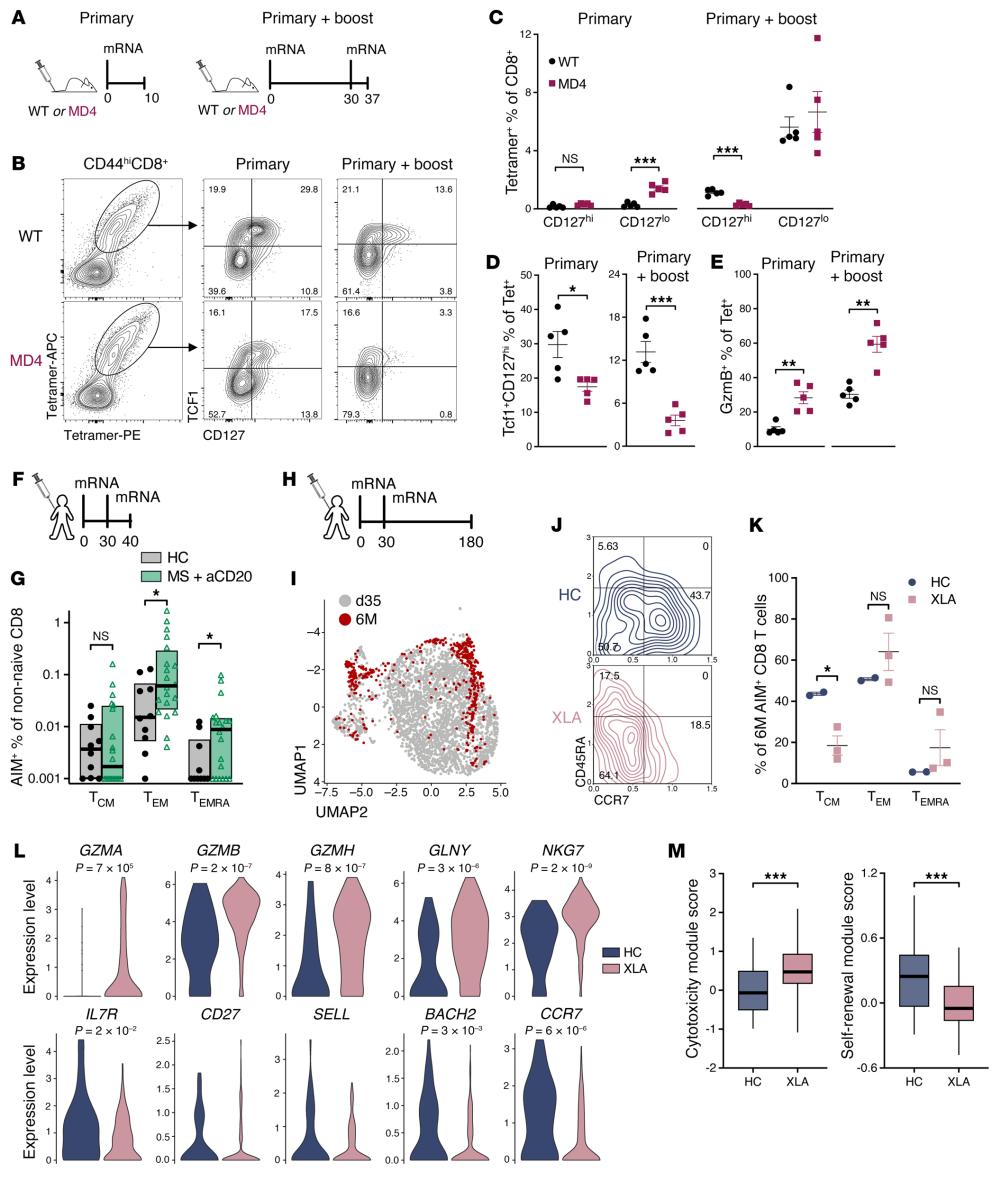
B cells limit effector CD8^+^ T cell expansion following mRNA LNP vaccination, preserving memory pool. (**A**–**E**) Mice were given either a primary mRNA LNP vaccination only (left) or a primary vaccine followed by a booster 30 days later (right). (**A**) Experimental schematic. (**B**) Representative dual tetramer staining for CD44^hi^ CD8^+^ T cells (left) and CD127 × TCF1 plots for tetramer^+^ cells after primary-only (middle) or primary-plus-boost (right) vaccination. (**C**) Spleens were assessed for the percentage of tetramer^+^CD127^hi^ cells and tetramer^+^CD127^lo^ cells out of total CD8^+^ T cells. (**D**) Percentage of TCF1^+^CD127^hi^ cells within tetramer^+^ cells. (**E**) Percentage of tetramer^+^ cells positive for granzyme B. Data shown are means ± SEM, *n* = 5 mice per group, representative of 2 experiments. Significance was defined by t tests; **P < 0.01, ***P < 0.001. (**F** and **G**) Healthy control (HC) subjects or patients with MS receiving anti-CD20 antibody therapy were assessed for antigen-specific T cells 10–12 days after an mRNA LNP COVID-19 vaccine boost. (**F**) Experimental schematic. (**G**) Antigen-specific cells were quantified as the percentage of non-naive CD8^+^ T cells positive for the activation-induced markers (AIMs) 4-1BB and IFN-γ and divided into T_CM_ (CD45RA^−^CD27^+^CCR7^+^), T_EM_ (CD45RA^−^CD27^−^CCR7^−^ plus CD45RA^−^CD27^+^CCR7^−^), and T_EMRA_ (CD45RA^+^CD27^−^CCR7^−^) subsets (boxes, 25th–75th percentile; horizontal lines, median). Significance was defined by Mann Whitney tests; *P ≤ 0.05. (**H**–**M**) HC subjects or XLA patients were vaccinated, and blood was taken 6 months after the first vaccine dose for single-cell sequencing. (**H**) Experimental schematic. (**I**) UMAP visualization of AIM^+^ (4-1BB^+^CD69^+^) CD8^+^ T cells highlighting the cells from 6 months after vaccination. (**J**) Contour plots of CITE-Seq protein data for CD45RA and CCR7 from HC subjects (top) and XLA patients (bottom). (**K**) Percentages of T_CM_, T_EM_, and T_EMRA_ were analyzed using 2-tailed Mann-Whitney tests; *P ≤ 0.05. (**L**) Violin plots of genes associated with cytotoxicity and memory. (**M**) Module scores for cytotoxicity and self-renewal gene signatures as box plots (vertical lines, minimum/maximum; boxes, 25th–75th percentile; horizontal lines, median) were analyzed using 2-tailed Mann-Whitney tests; **P < 0.01, ***P < 0.001.

We previously demonstrated that the impact of B cells on CD8^+^ T cell responses to vaccination was independent of the antigen, adjuvant formulation, and route of administration (1). Here, we aimed to investigate whether this holds true for mRNA lipid nanoparticle (LNP) vaccination, which has demonstrated obvious success in the clinic and has a heightened inflammatory profile compared with most other non-virus-based vaccines. We vaccinated mice with LNPs formulated similarly to SARS-CoV-2 mRNA-based vaccines. In these experiments, we assessed mice either at the peak of the T cell response after a single vaccination or after a primary vaccine followed by a secondary boost (Figure 5A and Supplemental Figure 5A). After a single vaccination, we observed no difference in the abundance of CD127^hi^ memory-phenotype CD8^+^ T cells. However, we found significantly more CD127^lo^ terminal effector cells in MD4 mice. After 2 vaccine doses, MD4 mice exhibited a specific defect in CD127^hi^ T cells, while the terminal effector cell numbers were equivalent between groups (Figure 5, B and C). Additionally, after both vaccination schedules, more T cells from WT mice coexpressed TCF1 together with high CD127 and fewer were positive for granzyme B compared with T cells from MD4 mice (Figure 5, D and E). Altogether, these results demonstrate the substantial influence that B cells exert on the response of naive CD8^+^ T cells to different vaccine formulations.

As noted above, recent reports on SARS-CoV-2 vaccination in humans have shown seemingly opposite results (11, 12). In agreement with these findings, we also found that the primary CD8^+^ T cell response to the mRNA LNP COVID vaccine was increased early after vaccination in MS patients with the lowest frequency of circulating B cells (13). However, this was entirely accounted for by an increase in terminal effector cells — specifically, effector memory (T_EM_) and effector memory cells reexpressing CD45RA (T_EMRA_)— rather than central memory (T_CM_) cells associated with long-lived immunity. Because this between-group comparison was not reported directly by Bar-Or and colleagues (11), we analyzed their publicly available data, taking care to gate the cells in the same manner as in the original publication (Supplemental Figure 5B). The data from the 3 originally reported effector subsets T_EM1–3_ were combined as T_EM_ for simplicity. Similar to what we found in our own study (11), at 10–12 days after a second COVID vaccine, there was no difference in the frequencies of activation-induced marker–positive (AIM^+^) T_CM_-phenotype cells but CD8^+^ T cells were increased for the terminally differentiated T_EM_- and T_EMRA_-phenotype cells in patients with MS receiving anti-CD20 therapy (Figure 5, F and G). At 25–30 days after vaccination, T_CM_ cells were undetectable for all but a few patients, and a trend toward increases in T_EM_ and T_EMRA_ cells remained but was insignificant (Supplemental Figure 5C). Thus, the increases Bar-Or and colleagues reported for MS patients treated with anti-CD20 at these early time points after vaccination were also limited to increases in terminal effector cell populations.

Only limited data exist regarding the long-term CD8^+^ T cell memory maintenance after COVID vaccination in the context of depleted or absent B cells. Buggert and colleagues used CITE-Seq (cellular indexing of transcriptomes and epitopes by sequencing) and single-cell RNA-Seq to analyze the phenotype and transcriptome of CD8^+^ T cells from X-linked agammaglobulinemia (XLA) patients (lacking functional B cells) and healthy controls 35 days and 6 months after the initial vaccine dose. They found that antigen-specific T cells from XLA patients exhibited increases in genes associated with terminal effector differentiation (14). We reanalyzed their 6-month time point sequencing data (Figure 5, H and I, and Supplemental Figure 5, D and E) taking advantage of the CCR7 and CD45RA CITE-Seq antibodies to determine the proportion of T_CM_, T_EM_, and T_EMRA_ cells at this late time point. T_CM_ cells represented a significantly smaller proportion of the AIM^+^ cells from XLA patients than from healthy controls (Figure 5, J and K), consistent with the mouse mRNA LNP vaccine data (Figure 5, A–E) and the memory phenotyping data we reported previously (1). The terminally differentiated T_EM_ and T_EMRA_ subsets showed a corresponding trend toward increased proportions in XLA patients. An increase in the expression of genes associated with effector function and terminal differentiation, including *PRF1*, *IFNG*, *GNLY*, *GZMB*, and *GZMH*, was noted in the original publication and could be summarized as a cytotoxic score (14). This score was significantly higher in CD8^+^ T cells derived from XLA patients, which we confirmed in our analyses (Figure 5, L and M). We further expanded these findings by evaluating the expression of genes associated with the self-renewal program, such as *IL7R*, *CD27*, *SELL*, *BACH2*, and *CCR7*. The expression of this self-renewal program was increased in healthy control cells compared with those from XLA patients (Figure 5L). Consequently, the self-renewal module score was significantly lower for the cells from XLA patients compared with healthy controls, demonstrating the impact of B cells on CD8^+^ T cell responses following COVID vaccination in humans (Figure 5M). Collectively, these data indicate that the expansion of terminal effector cells in the absence of a functional B cell compartment comes at the expense of memory-fated cells. Consistent results have been observed with adjuvanted subunit vaccines, replication-deficient viral vaccine vectors, multiple infections, and now mRNA LNPs. We conclude that B cells promote the formation of memory-fated CD8^+^ T cell responses to both vaccination and infection.

### FOXO1-haploinsufficient CD8^+^ T cells closely resemble those deprived of B cell help.

**Figure 6:**
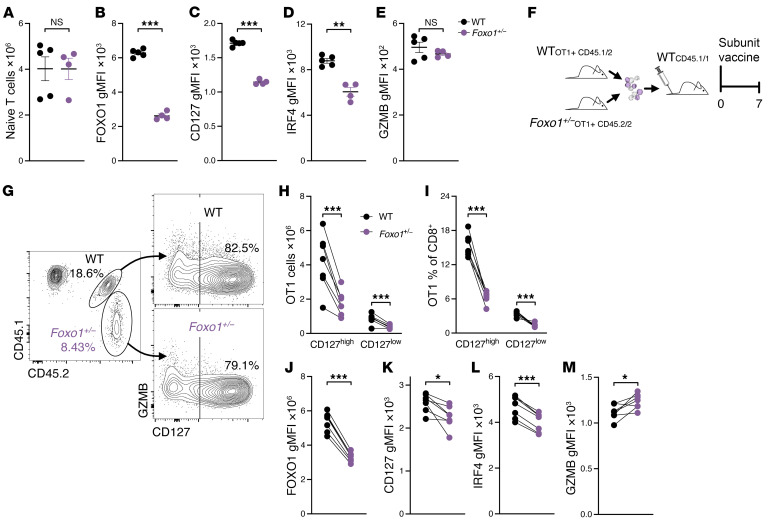
FOXO1-haploinsufficient CD8^+^ T cells closely resemble those deprived of B cell help. (**A**–**E**) Naive CD8^+^ T cells from unmanipulated *Foxo1^fl/WT^* E8I-Cre^+^ mice (*Foxo1^+/−^*) were compared with naive CD8^+^ T cells from unmanipulated Cre^–^ (WT) control mice. (**A**) The total number of splenic naive (CD44^lo^) CD8^+^ T cells was calculated for WT and *Foxo1^+/−^* mice. These cells were then assessed for the gMFI of FOXO1 (**B**), CD127 (**C**), IRF4 (**D**), and granzyme B (**E**). (**F**–**M**) OT1 T cells from congenically distinct *Foxo1^+/−^* and control mice were transferred into WT recipients, which were immediately immunized with the combined subunit vaccine. Seven days later, spleens were analyzed by flow cytometry. (**F**) Experimental schematic. (**G**) Representative staining for CD45.1 and CD45.2 (left) to identify OT1 cell genotype and CD127 staining on these cells (right). These data were used to calculate the number (**H**) and percentage (**I**) of responding OT1 T cells from each genotype. These cells were then assessed for the gMFI of FOXO1 (**J**), CD127 (**K**), IRF4 (**L**), and granzyme B (**M**). Data shown are means ± SEM, *n* = 4–5 mice per group, representative of 2 experiments. Significance was defined by t tests (**A**–**E**) or paired t tests (**H**–**M**); *P < 0.05, **P < 0.01, ***P < 0.001.

Our findings support the hypothesis that B cells promote naive CD8^+^ T cell homeostasis and memory formation by helping maintain FOXO1 levels. Though previous studies have shown that the complete absence of FOXO1 leads to severe defects in naive CD8^+^ T cell maintenance (31, 32) and memory formation (19, 23), the question remained whether partial reductions in FOXO1 would similarly affect T cell function. To address this, we generated CD8-specific FOXO1-haploinsufficient mice (*E8I-Cre^+^* *Foxo1^WT/fl^*, referred to as “*Foxo1^+/−^*”). Naive CD8^+^ T cell numbers were normal in these mice (Figure 6A), and their FOXO1 expression was about half that of E8I-Cre^–^ controls (“WT”), as expected (Figure 6B). This corresponded with a reduction in CD127 levels (Figure 6C). Interestingly, IRF4 expression was lower in *Foxo1^+/−^* cells (Figure 6D), which contrasts with our mouse data (Supplemental Figure 2C) but aligns with the results observed for naive CD8^+^ T cells from B cell–depleted patients (Figure 2G). Despite this, granzyme B expression remained normal in *Foxo1^+/−^* cells (Figure 6E), and in vitro culture with IL-7 revealed no survival defect over 7 days (Supplemental Figure 6), indicating that partial loss of FOXO1 reproduces some, but not all, aspects of the effector-skewing seen in B cell–deficient environments. When we cotransferred *Foxo1^+/−^* OT1 and WT OT1 cells into WT recipient mice and immunized them with the combined-adjuvant protein subunit vaccine, we found significantly fewer *Foxo1^+/−^* cells within both the CD127^hi^ and CD127^lo^ populations compared with WT cells (Figure 6, F–I). As with the naive T cells, FOXO1 levels in the activated *Foxo1^+/−^* cells were about half that of controls (Figure 6J), and CD127 expression within the *Foxo1^+/−^* CD127^hi^ population was also reduced (Figure 6K). IRF4 expression remained lower in *Foxo1^+/−^* cells (Figure 6L), while granzyme B was elevated (Figure 6M), demonstrating a shift toward terminal differentiation. These results indicate that reduced FOXO1 activity, similar to that observed in CD8^+^ T cells from B cell–deficient environments, directs T cell effector-skewing and reduced memory potential.

## Discussion

B cell–depleting therapies are increasingly used to treat cancer and autoimmune diseases. These treatments commonly lead to B cell aplasia and sometimes result in hypogammaglobulinemia and frequent infections (45, 46). Understanding how these treatments affect vaccine responses and immunity to infections is vital. Unsurprisingly, the induction of humoral immunity by most vaccines is impaired (47). Remarkably, mouse models indicate that B cell aplasia also leads to impaired CD8^+^ T cell immunity following both vaccination and infection. Here, we clarified conflicting reports on the role of B cells in influencing CD8^+^ T cell responses following vaccination in humans and expanded upon these findings, revealing a significant role for B cells in programming naive CD8^+^ T cells, which profoundly affects their fate following vaccination.

Patients undergoing B cell–depleting therapies can still generate T cell responses to vaccines, although the CD8^+^ T cell responses are altered. Some studies have reported diminished responses following influenza (2, 7, 8), tetanus toxoid (9), and SARS-CoV-2 (10, 48) vaccinations, while other studies report seemingly opposite results (11, 12). Our recent study (13), together with the reanalysis of published data sets (11) presented here, found that the enhancement in CD8^+^ T cell responses reported for MS patients depleted of B cells was limited to terminal effector cells. This was consistent with the reanalysis of single-cell sequencing data from XLA patients (14) lacking functional B cells from birth, which revealed significantly reduced central-memory-phenotype CD8^+^ T cells 6 months after SARS-CoV-2 vaccination. The antigen-specific CD8^+^ T cells present at this late stage exhibited a more cytotoxic gene signature but a reduced self-renewal/memory gene signature. Thus, we have shown that humans lacking B cells exhibit a similar defect in their ability to generate CD8^+^ T cell memory following vaccination as observed in mice. Data from Gaiha and colleagues, demonstrating that rituximab-treated MS patients have lower preexisting antiviral CD8^+^ T cell immunity as measured by stimulation of PBMCs with a combination of CMV, EBV, and flu (CEF) peptides (12), further suggest that this deficiency extends to generating long-lived memory following infection.

We used the MD4 mouse model to study CD8^+^ T cells lacking B cell help because this model reproduces findings from μMT^−/−^ and B cell–depleted mice without the potentially confounding influence of a defective lymphoid architecture (1). Notably, depleting B cells in MD4 mice did not rescue the CD8^+^ T cell phenotype but instead enhanced the effector-skewing of CD8^+^ T cells and reduced their overall magnitude. This indicates that the observed phenotype is not due to an aberrant gain of function by MD4 B cells but rather reflects a loss of WT B cell function(s) responsible for promoting a memory phenotype.

Until now, very little was known about how or when B cells exert their influence over CD8^+^ T cells, representing a major gap in understanding. Transcriptomic analysis we performed of naive CD8^+^ T cells from WT and MD4 mice uncovered a role for B cells in supporting the FOXO1-governed homeostatic quiescence of these cells. Protein expression analysis from patients receiving B cell–depleting therapies showed similar results, indicating that this mechanism operates in both mice and humans. Naive CD8^+^ T cells were reduced in number in MD4 mice, as has been reported for μMT^−/−^ mice and in humans following B cell depletion (49). In vivo and in vitro assays indicated that this reduction stems from compromised survival. Cotransfer experiments showed that the phenotype of naive CD8^+^ T cells from MD4 mice was stable, and their impaired ability to generate memory was significantly influenced by the B cell environment in which they developed. Additionally, we demonstrated that reducing FOXO1 levels by half in CD8^+^ T cells — similar to levels we see in B cell–deficient environments — was sufficient to largely recapitulate the effector-skewing in CD8^+^ T cells before and after vaccination seen in various models and human patients lacking B cells. This indicates that B cell support of FOXO1 expression in CD8^+^ T cells is crucial for sustaining quiescence and promoting memory formation.

These findings not only shed light on the productive interactions between B cells and CD8^+^ T cells but also provide insight into FOXO1 biology. FOXO1 has been previously shown to maintain naive T cell homeostasis (31, 32) and is critical for the development and maintenance of memory T cells (19). While primary T cell responses following LM infection proceed relatively unabated in the absence of FOXO1, the responding cells display an exclusively effector phenotype (19, 23). In contrast, FOXO1 deficiency significantly impacts the magnitude of CD8^+^ T cells after subunit vaccination (23), likely because a vast majority of cells responding to subunit vaccination in WT mice display a memory phenotype (CD127^hi^TCF1^+^). Here, we show that the role of FOXO1 in CD8^+^ T cell memory development is analog rather than digital; reducing FOXO1 levels by 50% results in a proportionately reduced memory-phenotype response. In the context of defective B cell help, we observed consistently defective memory alongside numbers of short-lived effector cells that were either roughly equivalent (subunit vaccination, mRNA LNP boost, vaccinia virus), reduced (LCMV), or even enhanced (mRNA LNP primary) compared with controls, depending on the insult and timing. This explains the seemingly contradictory clinical evidence showing both reduced (2, 7–10) and enhanced (11, 12) CD8^+^ T cell responses in B cell–depleted patients. These patients may initially produce normal or exaggerated numbers of T cells depending on their capacity to generate the inflammatory environment necessary to facilitate terminal differentiation in the responding T cells. However, given the exclusive enhancement of terminal-effector-phenotype cells we found in our analyses of human samples from several studies (11, 13, 14), any enhanced T cell responses are predicted to be short-lived and provide limited long-term protection.

These findings have major implications for patients undergoing B cell–depleting therapies and those with congenital B cell deficiencies, such as common variable immunodeficiency and XLA, who rely heavily on T cell immunity for protection from infection. Several important questions arise: Can patients stop treatment before vaccination to restore normal T cell responses, or will they need extended recovery time from B cell depletion for new naive T cells to develop? Will more frequent vaccine boosters help bolster long-term protection, or will they deplete fledgling memory reserves to create short-lived populations? Could exaggerated effector T cell responses lead to increased immunopathology? And finally, are certain vaccine platforms more or less likely to exacerbate the dual effects of elevated effector responses and depleted memory formation? Further investigations are essential to address these questions and to elucidate the mechanisms by which B cells regulate FOXO1 in CD8^+^ T cells, potentially leading to novel therapeutic strategies for affected patients.

## Methods

### Sex as a biological variable

Human studies included both male and female participants as documented in Supplemental Table 1. Female participants were more prevalent, consistent with the higher incidence of MS in women. Mouse studies were conducted in both males and females, and similar findings were observed for both sexes.

### Study design

Experimentation on human blood samples was performed in a blinded manner, in which individuals were identified by a unique alphanumeric identifier, and personally identifiable information was not available to personnel carrying out the experiments. Unblinding occurred after analyses. Power calculations for human studies were not performed, because of limited data available in advance on which to base the calculations. Since higher variability was expected in human subject samples, all CAR T cell patient samples that met the inclusion and exclusion criteria outlined below were included, and a relatively high number of healthy control subject and MS patient samples were collected (*n* = 20 per group) compared with what was used for mouse studies. If more than about 5 mice per group were required to obtain a significance level of *P* ≤ 0.05, the difference between experimental and control groups was deemed too small and/or too variable to be considered biologically relevant. Mouse experiments were typically repeated once, as indicated in figure legends.

### Mice

WT (C57BL/6J), congenic CD45.1 B6 (B6.SJL-*Ptprc^a^* *Pepc^b^*/BoyJ), OVA-specific TCR-transgenic OT1 [C57BL/6-Tg(TcraTcrb)1100Mjb/J], μMT^−/−^ (B6.129S2-*Ighm^tm1Cgn^*/J), and MD4 [C57BL/6-Tg(IghelMD4)4Ccg/J] mice were originally obtained from The Jackson Laboratory and subsequently bred in-house at the University of Colorado. MD4 mice, originally generated on the C57BL/6J background (50), were bred as hemizygous MD4 × C57BL/6J at our facility and backcrossed here for more than 10 additional generations, and hemizygous MD4 mice were compared with cohoused WT littermate controls. *Foxo1^fl/fl^* mice, a gift from Stephen Hedrick (UCSD, San Diego California, USA), were crossed to E8I-Cre [C57BL/6-Tg(Cd8a-cre)1Itan/J] mice or OT1^+^ E8I-Cre mice to generate animals with CD8^+^ T cell–specific *Foxo1* haploinsufficiency. Experiments were performed in 6- to 12-week-old male and female mice.

### CAR T cell patient inclusion and exclusion criteria

Included patients had received anti-CD19 CAR T cell therapy for the treatment of B cell malignancies, e.g., B cell acute lymphoblastic leukemia, diffuse large B cell lymphoma, mantle cell lymphoma, or follicular lymphoma; had achieved complete remission following anti-CD19 CAR T cell therapy; and had a PBMC sample available at a time point when they were still in remission (time points lacking B cell aplasia were excluded) and at least 4 weeks after the initiation of therapy (the latest time point in which B cell aplasia and remission were both noted was preferred). Patients were excluded if they received additional immunotherapies, such as checkpoint inhibitors, after the initiation of anti-CD19 CAR T cell therapy but prior to PBMC sample collection; received chemotherapy or radiation therapy after the initiation of CAR therapy, not including whatever lymphodepleting regimen was used directly before the infusion of CAR T cells; had a history of autoimmune disorders or developed autoimmune complications after CAR T therapy; had infections documented up to 7 days before PBMC sample collection; received a second dose of anti-CD19 CAR T cell therapy or other T cell therapies prior to PBMC sample collection; received allogeneic stem cell transplantation prior to sample collection; received immunosuppressive medications, such as corticosteroids or calcineurin inhibitors, within 3 weeks prior to PBMC sample collection; were pregnant at the time of PBMC sample collection; or had been diagnosed with non–B cell malignancies, excluding non-metastatic squamous or basal cell carcinomas, unless the patient was more than 5 years out from any prior malignancy with no evidence of disease.

### Immunizations and infections

#### Combined-adjuvant protein subunit vaccine.

Mice were immunized via tail vein injection (i.v.) with 150 μg of whole chicken ovalbumin (OVA; MilliporeSigma) plus 40 μg poly(I:C) (InvivoGen) and 40 μg anti-CD40 (clone FGK4.5, Bio X Cell). OVA (MilliporeSigma) protein was detoxified by phase separation (51) and was lipopolysaccharide-free as determined by a Limulus assay. Vaccines were made immediately before immunization.

#### Lipid nanoparticle vaccine.

The full-length DNA sequence of OVA with optimized 5′ and 3′ UTRs and in-frame 3× FLAG tag was synthesized in a pTwist Kan High Copy plasmid vector (Twist Bioscience). CleanCap AG capped mRNA, including N1-methylpseudouridine-5′-triphosphate substitutions, was produced by T7 RNA polymerase transcription from linearized plasmid templates and subsequently column-purified and enzymatically polyadenylated with *E.* *coli* poly(A) polymerase. Column-purified mRNA transcripts were incorporated into LNPs composed of Precision NanoSystems Neuro9 lipid mix (catalog NWS0001) on a microfluidic LNP mixer (Precision NanoSystems Spark) and dialyzed twice with PBS (Corning). Encapsulation efficiency and RNA content of LNPs were quantified using the RiboGreen RNA Assay Kit (Thermo Fisher Scientific), a dye-binding assay, after detergent-based dissolution of the LNPs. Concentrations of 50–100 ng/mL were used for i.v. administration. A primary dose of OVA-LNPs containing 2 μg of mRNA was followed by a matching boost 1 month later. Spleens were harvested and processed for flow cytometry 7 days after the boost.

LCMV Armstrong was propagated on baby hamster kidney 21 cells (BHK21 [C-13], ATCC CCL10), and viral titers were determined using a plaque assay with Vero-E6 cells (ATCC CRL-1586), as previously described (52). LCMV infections were performed by administration of 2 × 10^5^ plaque-forming units (PFU) of viral stocks diluted in PBS via intraperitoneal injection. Vaccinia virus Western Reserve (VV-WR) was grown and titered using Vero cells. VV-WR infections were performed by administration of 1 × 10^7^ PFU intravenously.

### Mouse CAR T cell experiments

CAR constructs included an anti-CD19 clone, 1D3 ScFv, with a CD28 hinge/transmembrane domain, a CD28 costimulatory domain, and the CD3 ζ chain (53) with all 3 immunoreceptor tyrosine-based activation motifs intact, and P2A sequence preceding a truncated human EGFR, for detection. After synthesis (GeneArt, Thermo Fisher Scientific), constructs were cloned into γ-retroviral transfer plasmids. Retroviral vectors encoding each CAR were produced by transient transfection of the Platinum-E cell line, which stably expresses gag, pol, and ectotrophic env genes, using Lipofectamine 3000 (Life Technologies) with plasmids encoding the CAR constructs. Supernatants were collected 48 hours after transfection. Four days before adoptive transfer (day –4), T cells were extracted from CD45.1^+^ mouse splenocytes using an EasySep Mouse T cell isolation kit (StemCell Technologies), activated using anti-CD3/CD28 beads (Life Technologies) at a 1:1 bead/cell ratio, and cultured in complete RPMI medium with recombinant human IL-2 (rhIL-2) (40 IU/mL; R&D Systems) and rhIL-7 (10 ng/mL; R&D Systems), at 1 × 10^6^ cells/mL. The next day (day –3), retroviral supernatant was added to RetroNectin-coated (Takara Biosciences) 6-well plates and spun at 2,000*g* at 32°C for 2–3 hours. Supernatant was then removed, and activated T cells were added to the wells at 1.67 mL/well. On day –1, beads were removed, and T cells were resuspended at 1 × 10^6^/mL in fresh medium with rhIL-2 (40 IU/mL) and rhIL-7 (10 ng/mL). Transduction efficiency was analyzed by EGFR expression. Control cells used in these experiments were activated in the same manner but were not retrovirally transduced. Mice received 1 × 10^6^ CAR T cells or control cells, adjusted based on transduction efficiency (typically ≥80%), 3 hours after being non-lethally irradiated (3 Gy). Mice were bled 10, 14, and 31 days later, then vaccinated with the combined subunit vaccine, and spleens were harvested 7 days later for analysis by flow cytometry.

### Human CAR T cell therapy

The human CAR T cell construct is composed of the short-chain variable regions of the anti-CD19 monoclonal antibody FMC63, with a TNFRSF19-derived transmembrane domain and a 4-1BB costimulatory and a CD3-ζ signaling domain. CD19 CAR T cells were manufactured using the CliniMACS Prodigy T Cell Transduction Process, with the CD3/CD28 TransAct reagent (Miltenyi) allowing for highly automated production, with IL-7 and IL-15 used for T cell expansion for 8–12 days. Patients were infused with an average dose of 1 × 10^8^ CAR T cells.

### RNA processing and differential expression analysis

Naive CD8^+^ T cells from 5 WT and 5 MD4 10-week-old female mice were individually sorted to greater than 99% purity on a BD FACSAria (Becton Dickinson), gating on CD8^+^CD19^−^B220^−^CD44^lo^ live single cells, after enrichment using a magnetic CD8^+^ T cell isolation kit (BioLegend). RNA was prepared using a PureLink RNA Mini Kit (Thermo Fisher Scientific). mRNA quality was assessed using NanoDrop (Thermo Fisher Scientific) and RNA ScreenTape Analysis on an Agilent 2200 TapeStation; all 10 samples had RNA integrity number equivalent (RINe) scores greater than 9, indicating negligible RNA degradation. The Universal Plus mRNA-Seq library preparation kit with NuQuant (Tecan) was used with an input of 100 ng of total RNA to generate RNA-Seq libraries. Paired-end sequencing reads of 150 bp were generated on a NovaSeq 6000 (Illumina) sequencer at a depth of about 40 million clusters/80 million paired-end reads per sample at the University of Colorado Anschutz Medical Campus Genomics Core. Raw sequencing reads were de-multiplexed using bcl2fastq (Illumina). Quality of reads was assessed before and after trimming using FastQC v0.1.3 (54). Illumina universal adapters were removed, bases were trimmed if the Phred score was less than 24, and any reads after trimming that were fewer than 20 bp in length were discarded using Cutadapt v4.2 (55) under Python v3.11.4. Reads were aligned and quantified to the mm10 reference genome using Rsubread v2.12.3 (56). The quality of the alignments was assessed using PicardTools v2.27.4 (57). Differential gene expression was performed using DESeq2 v1.38.3 (58). Differentially expressed genes were defined as genes with an adjusted *P* value less than 0.05. ChEA (30) 2022 dataset analysis was performed by inputting of this mouse gene list into the Enrichr web interface (https://maayanlab.cloud/Enrichr/) (59) and sorting of the table of associated transcription factors by combined score, which integrates the adjusted *P* values and odds ratios.

### Adoptive transfers

CD8^+^ T cells were magnetically purified (BioLegend) from WT or MD4 mice to greater than 95% purity. For naive T cell survival experiments (Figure 3B), 2.5 million WT plus 2.5 million MD4 congenically distinct (based on CD45.1/2, as indicated) CD8^+^ T cells were cotransferred to recipient mice by tail vein injection. For immunization experiments (Figure 4), 200 WT plus 200 MD4 OT1 T cells or 2 million WT plus 2 million MD4 (non-transgenic) CD8^+^ T cells (Supplemental Figure 4) were cotransferred immediately before immunization or 1 month before immunization, respectively.

### In vitro survival and proliferation assays

Mouse CD8^+^ T cells were purified by magnetic enrichment (BioLegend), labeled with CellTrace Violet (CTV; Invitrogen), and plated in RPMI 1640 containing 10% FBS, 10 mM HEPES, 0.1 mM beta mercaptoethanol, 0.1 mM non-essential amino acids, 0.1 mM sodium pyruvate, 2 mM l-glutamine, and penicillin-streptomycin (“complete medium”) at 100,000 cells per well in a 96-well, round-bottom plate (Corning Costar). To assay survival, cells were incubated with either 5 ng/mL human IL-7 (R&D Systems) or no additional cytokines. Wells were counted immediately after plating and again 2 and 7 days later. For proliferation assays, cells were plated on 96-well plates precoated with 1.25, 2.5, 5, or 10 μg/mL of anti-CD3 antibody (145-2C11, Tonbo) overnight at 4°C, then washed twice with complete medium. Cells were plated with soluble anti-CD28 (37.51, BioLegend) at 1 μg/mL for 3 days before assessment of division by CTV dilution.

### In vivo proliferation assays

#### Lymphopenia-induced proliferation.

Recipient WT (CD45.2/2) or MD4 (CD45.2/2) mice were irradiated (6 Gy). The next day, 100,000 WT plus 100,000 MD4 purified, congenically distinct OT1 cells were labeled with proliferation dye, cotransferred (see above), and given 10 days to undergo lymphopenia-induced proliferation before flow cytometry was performed on harvested spleens.

#### Vaccine-induced proliferation.

Magnetically enriched, congenically distinct OT1 T cells were purified from the spleens of WT and MD4 mice and mixed 1:1. A total of 50,000 cells were then cotransferred into recipient mice by tail vein injection (see above) followed by immediate immunization with the combined-adjuvant subunit vaccine. On day 3 after vaccination, mice were injected i.v. with EdU at 20 mg/kg. Three hours later, spleens were harvested and assessed for EdU incorporation by flow cytometry using a Click-iT AF594 assay kit (Molecular Probes).

#### Mouse sample processing and flow cytometry.

Spleen single-cell suspensions or whole blood (collected by tail vein puncture into 5 mM EDTA in HBSS), as indicated, were subjected to ACK red blood cell lysis and counted using a Vi-Cell automated cell counter (Beckman Coulter). Cells were then incubated with anti-CD16/32 (clone 2.4G2; hybridoma supernatant) and stained with tetramer at 37°C for 30 minutes in complete medium. Kb-SIINFEKL, Kb-B8R, Db-GP33, Db-NP396, and Db-GP276 tetramers were provided by the NIH Tetramer Core. After tetramer staining (if applicable), samples were washed and incubated with surface antibodies/viability dye for 10 minutes at 37°C in complete medium, including different combinations of CD8A (clone 53-6.7), CD19 (1D3; BD Biosciences), CD44 (IM7; Tonbo), CD45 (clone 30-F11), CD45.1 (clone A20), CD45.2 (clone 104), CD49d (clone R1-2), CD62L (clone MEL-14), CD122 (clone TM-b1), CD127 (clone A7R34), human EGFR (clone AY13), KLRG1 (clone 2F1/KLRG1), TCRβ (clone H57-597), and Ghost Dye Red 780 (fixable viability dye, Tonbo), all from BioLegend unless otherwise noted. For transcription factor analyses, cells were surface-stained, then fixed and permeabilized using Tonbo Foxp3 fixation/permeabilization buffers. After fixation and permeabilization, cells were stained for EOMES (Dan11mag, eBioscience), IRF4 (Irf4.3E4, BioLegend), granzyme B (NGZB, Invitrogen), FOXO1 (C29H4, Cell Signaling Technology), T-bet (4B10, BioLegend), and TCF1 (C63D9, Cell Signaling Technology). Flow cytometry data were acquired on a 4-laser (405, 488, 561, and 638 nm) CytoFLEX S flow cytometer (Beckman Coulter), and analysis was performed using FlowJo (v10.10.0, BD Biosciences).

### Human sample processing and flow cytometry

Blood of patients with MS and healthy control blood were collected as part of standard-of-care procedures in two 15 mL glass Vacutainers (BD Biosciences) containing 1.5 mL 3.8% sodium citrate solution. Both Vacutainers were then pooled together in a 50 mL Leucosep tube (Greiner Bio-One) filled with 15 mL Lymphoprep (StemCell). The Leucosep tube was then centrifuged at 1,800*g* for 15 minutes at half brake. CAR T cell patient samples were collected and processed similarly. Instead of the use of a Leucosep tube with Lymphoprep, blood was mixed 1:1 with DPBS, then overlaid on top of 15 mL of Ficoll in a 50 mL conical tube. Tubes were centrifuged for 15 minutes at 800*g* with no brake. After centrifugation, the PBMC buffy coats were treated in essentially the same manner. Buffy coats were transferred into a fresh 50 mL conical tube and diluted 1–1.5× by volume in DPBS. PBMCs were then centrifuged at 500*g* for 10 minutes. The supernatant was removed and washed in additional DPBS. Finally, the PBMC pellet was resuspended in FBS plus 10% DMSO, aliquoted, and immediately transferred to –80°C storage before transfer into liquid nitrogen.

Human PBMC samples were all thawed, stained, and run on the cytometer on the same day. Cells were rapidly thawed in a 37°C water bath, washed, resuspended in staining buffer, filtered through a 70 μm mesh (Fisher), placed on ice, and counted. Cells were stained with surface antibodies for 10 minutes at 37°C prepared in complete medium with Brilliant Stain Buffer (BD Biosciences), including CD8A-BUV395 (HIT8a), CD3-BUV496 (UCHT1), CD45RO-BUV563 (UCHL1), CD19-BUV661 (HIB19), CX3CR1-BUV805 (2A9-1), Ghost Dye Violet 510 (fixable viability dye, Tonbo), CD49d-BV605 (9F10, BioLegend), CCR7-BV711 (G043H7, BioLegend), CD127-BV785 (A019D5, BioLegend), CD4-B548 (SK3, Cytek), CTLA4-BB700 (BNI3), NKG2A-APC (REA110, Miltenyi Biotec), and NKG2D–APC/Fire 750 (1D11, BioLegend), all from BD Biosciences unless otherwise noted. For transcription factor analysis, after surface staining, cells were washed, then fixed and permeabilized using Foxp3/Transcription Factor Staining Buffers (Tonbo) for 20 minutes at room temperature. After fixation, cells were stained for p-AKT (S473)–biotin (D9E, Cell Signaling Technology), IRF4-VioB515 (REA201, Miltenyi Biotec), FOXO1-PE (C29H4, Cell Signaling Technology), p-ERK1/2 (T202/Y204)–PE/eF610 (MILAN8R, eBioscience), p–p38 MAPK (T180/Y182)–PE/Cy7, and granzyme A–R718 (CB9), all from BD Biosciences unless otherwise noted. Cells were washed, then stained for 10 minutes at room temperature with streptavidin-BV421, after which they were washed again. Flow cytometry data were acquired on a 5-laser (355, 405, 488, 561, and 640 nm) Aurora flow cytometer (Cytek). After unmixing of raw data on the cytometer, further analysis was performed using FlowJo (v10.10.0, BD Biosciences).

### Reanalysis of published healthy control versus MS plus anti-CD20 flow cytometry data

Compensated flow cytometry .fcs files for the AIM T cell analysis were downloaded from Cytobank (https://premium.cytobank.org/cytobank/experiments/378713) (11). Samples were matched to their time point and group based on the accompanying “experiment_378713_annotations.txt” file. Gating was performed as shown (Supplemental Figure 4, A–D). AIM^+^ CD8^+^ T cells were defined by dual expression of 4-1BB and intracellular IFN-γ. AIM^+^ percentage of non-naive values for the T_CM_, T_EM_, and T_EMRA_ subsets was calculated as “CD8-E megapool”–stimulated minus paired, unstimulated controls. Between-group comparisons were made for the 10–12 days after the boost (“T4”) and 25–30 days after the boost (“T5”) time points using 2-tailed Mann-Whitney tests, *P ≤ 0.05.

### Reanalysis of published healthy control versus XLA patient single-cell RNA sequencing data

Processed single-cell RNA-Seq data were downloaded from ArrayExpress (http://www.ebi.ac.uk/arrayexpress) using accession number E-MTAB-11845 (14). Expression data (gene, protein, and hashtag) were combined using the R package Seurat (v5.0.1) with the Read10X function. As in the original publication, cells were removed if they contained more than 7% of reads aligned to mitochondrial genes or expressed fewer than 700 genes or more than 5,700 genes. Transcript expression was normalized using the LogNormalize method implemented in Seurat’s NormalizeData function. Expression for antibody-derived tags (protein) and hashtag oligonucleotides (HTOs) was transformed using the centered log-ratio method. HTO demultiplexing was performed using Seurat’s HTODemux function. Gene expression values were scaled and centered using the ScaleData function (Seurat), and highly variable genes were selected using the Seurat vst algorithm. Clusters were identified using the FindNeighbors function with 30 dimensions and the FindClusters function with the Louvain algorithm. Dimensionality reduction was performed using the uniform manifold approximation and projection (UMAP) algorithm. CD8^+^ T cells were identified based on CD8A protein and *CD8A* gene expression (Supplemental Figure 5 D and E). CD4^+^ T cells and unconventional CD8^+^ T cells were removed using the following criteria: CD4^+^ T cells (CD4 protein^+^ and/or *CD4^+^* clusters), MAIT cells (*SLC4A10^+^* and *TRAV1-2*^+^ clusters), γδ T cells (*TRDC^+^* and *TRDV1^+^* clusters). Conventional CD8^+^ T cells were reclustered and separated into day 35 and 6-month time points based on their hashtags (Figure 5I). Because XLA patients were not equally represented, cells were downsampled to ensure equal representation before pairwise comparisons for individual genes and module scoring. Gene module scoring was performed using the AddModuleScore function (Seurat). A manually curated gene set for CD8^+^ T cell cytotoxicity was sourced from a study of total peripheral responses during COVID-19 (60), as it was for the original publication (*PRF1*, *IFNG*, *GNLY*, *NKG7*, *GZMB*, *GZMA*, *GZMH*, *KLRK1*, *KLRB1*, *KLRD1*, *CTSW*, *CCL5*, *CST7*). A manually curated gene set was used for CD8^+^ T cell self-renewal based on studies of memory T cell generation and maintenance (*IL7R*, *CD27*, *SELL*, *BACH2* [ref. 61], *CCR7*, *TCF7* [ref. 25], *CXCR4* [ref. 62], *NFKB1* [ref. 63], *NFKB2* [ref. 63], and *MYB* [ref. 64]). Pairwise comparisons and module scores were compared by the Mann-Whitney test implemented in the wilcox.test R function.

### Statistics

GraphPad Prism (v10.1.0) was used to generate graphs and for all statistical analyses other than those performed on the RNA sequencing data. Heatmaps were generated using Morpheus (Broad Institute). Figure legends detail the number of experimental replicates and *n* values. Unless noted, data shown are means ± SEM, and significance was defined by a 2-tailed, unpaired Student’s *t* test, where *P < 0.05, **P < 0.01, and ***P < 0.001.

### Study approval

Human samples were collected under the approved Colorado Multiple Institutional Review Board protocols 19-2807 and 18-2424 after written informed consent and used under the approved secondary use protocol 23-1309. Healthy human control subjects were enrolled at the University of Colorado School of Medicine neurology clinic, presenting with migraines but no known history of autoimmune disease. MS patients, enrolled at the same clinic, were selected retrospectively to fit into the 2 treatment groups compared here. CAR T cell patients were obtained from the Gates Institute Biobank (University of Colorado Anschutz Medical Campus). All experiments involving mice were conducted following protocols approved by the University of Colorado Institutional Animal Care and Use Committee according to guidelines provided by the Association for Assessment and Accreditation of Laboratory Animal Care (AAALAC).

### Data availability

All data associated with this study are present in the paper or the supplemental materials. Source data without protected health information will be made available upon request. Values for all data points in graphs are reported in the Supporting Data Values file. RNA sequencing data analyzed in Figure 2 were deposited in the NCBI’s Gene Expression Omnibus (GEO) repository. The data were assigned the following GEO accession numbers: GSE263132, GSM8186507, GSM8186508, GSM8186509, GSM8186510, GSM8186511, GSM8186512, GSM8186513, GSM8186514, GSM8186515, and GSM8186516.

## Author contributions

RMK and JK conceptualized the study. CM, MGM, RMK, and JK designed the experiments. CM, MGM, JC, MGH, MAD, and JK generated and analyzed the data. TMB, LG, and JK performed sequencing analyses. JC, ELL, JRH, MRV, and ALP contributed key reagents and methods. SS, TLB, and CM processed human samples and/or obtained clinical data. EWYH, MRV, ALP, and LG performed data evaluation and supervision. CM, RMK, and JK wrote the manuscript.

## Supplementary Material

Supplemental data

Supporting data values

## Version 1 04/17/2025

In-Press Preview

## Version 2 06/16/2025

Electronic publication
