# Supplementary material for: B cells shape naive CD8+ T cell programming
Source: J Clin Invest. 2025 Apr 17;135(12):e190106. doi: 10.1172/JCI190106 (PMC12165804; doi:10.1172/JCI190106)
Supplement: Supplemental data [file jci-135-190106-s117.pdf]

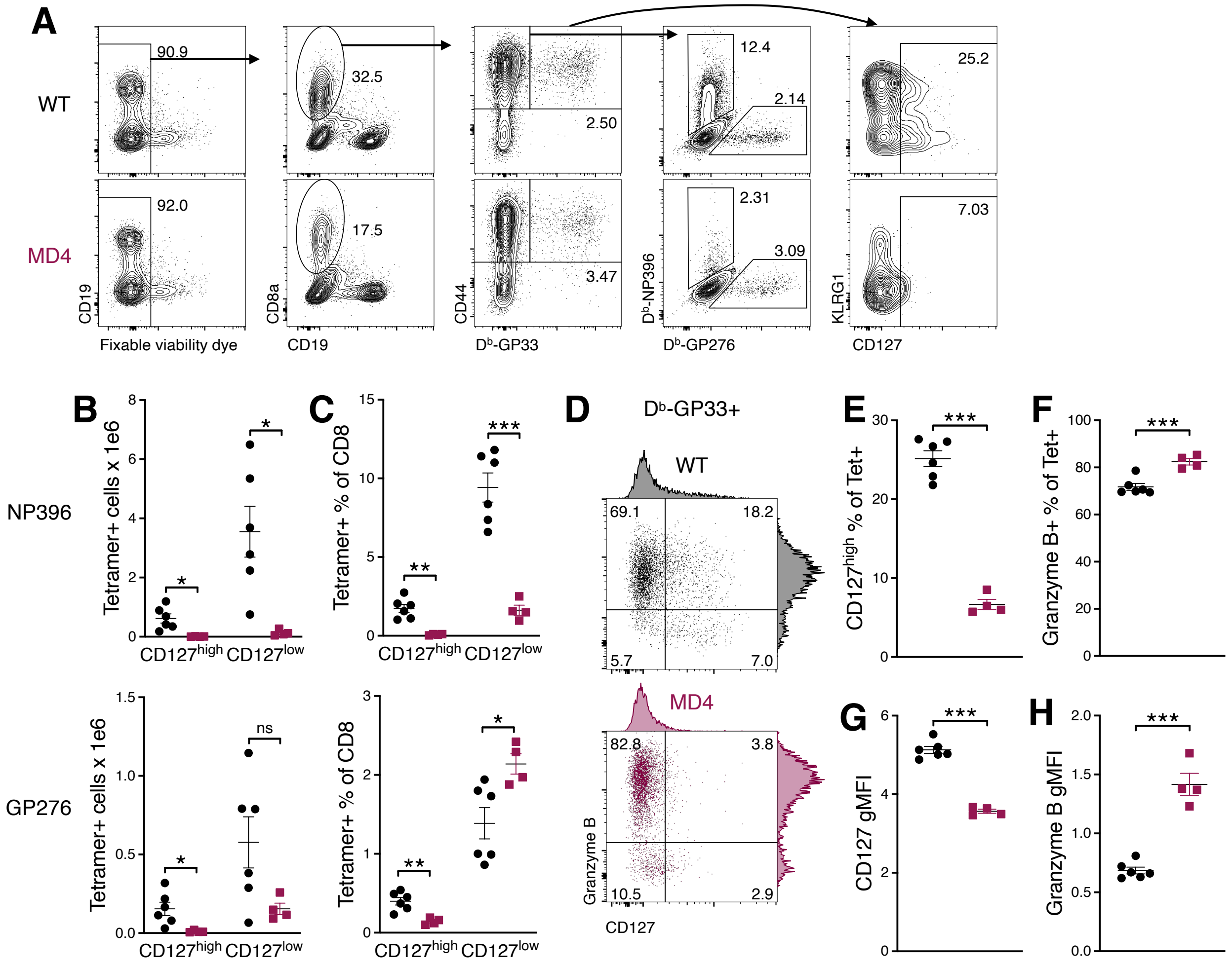

Figure S1. B cells promote memory-fated CD8 T cell responses to vaccination and infection. (**A**) Representative flow cytometry gating, pre-gated on single-cell lymphocytes. (**B-C**) Eight days after LCMV-Armstrong infection**,** spleens were assed for (**B**) the number of NP396 (top) or GP276 (bottom) tetramer+ cells, and (**C**) the percentage of CD8 T cells positive for tetramer. (**D**) Representative CD127 x granzyme B staining for GP33 tetramer+ cells. (**E-F**) The percentage of GP33 tetramer+ cells (**E**) expressing high CD127 and (**F**) positive for granzyme B. (**G**) The CD127 gMFI of CD127high cells. (**H**) The granzyme B gMFI of granzyme B+ cells.

Supplementary Table 1: Human subject demographics

Characteristic	Healthy control	CAR T therapy	MS anti-α4 integrin	MS anti-CD20
Median age, years (range)	40 (27-68)	50 (6-79)	46 (25-66)	49 (30-72)
Female sex, n (%)	18 (94.7)	2 (25)	14 (74)	17 (85)
Median BMI, (range)	27 (20-37)	25 (13-18)	29 (20-44)	28 (19-62)
Therapy, n (%)				
Natalizumab			19 (100)	
Rituximab				11 (55)
Ocrelizumab				9 (45)
anti-CD19 CAR		8 (100)		
Median time on therapy, months, (range)		9 (3-15)	82 (15-143)	25 (5-90)
Primary disease, n (%)				
Relapsing-remitting MS			18 (95)	20 (100)
Secondary-progressive MS			1 (5)	
B cell acute lymphoblastic leukemia (B-ALL)		3 (37.5)		
Diffuse large B-cell lymphoma (DLBCL)		2 (25)		
Follicular lymphoma (FL)		2 (25)		
Transformed lymphoma (TL)		1 (12.5)		

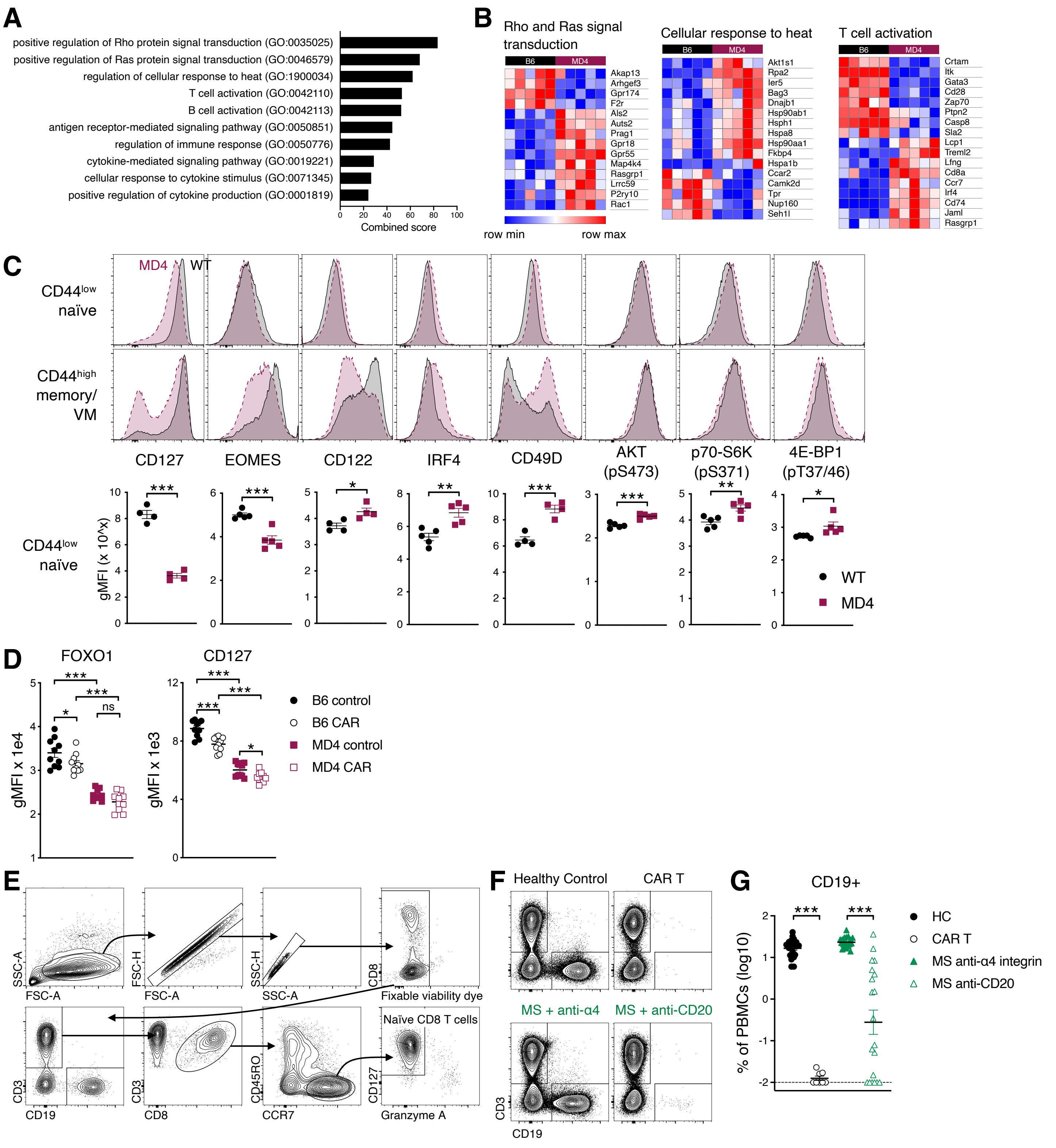

Figure S2. B cells shape naïve CD8 T cell programming, promoting FOXO1-mediated homeostasis in mice and humans. (**A**) GSEA was performed using the GO pathway gene sets. Depicted are the top 10 data sets based on combined score. (**B**) Heat maps for the top four gene sets, where the differentially-expressed genes associated with the Rho and Ras protein signal transduction gene sets were combined into one heat map. (**C**) Flow cytometry staining of CD8 T cells from WT and MD4 mice. Representative histograms are shown for CD44^{low} naïve (top panels) and for CD44^{high} memory and virtual memory (middle panels) cells. Plots of the gMFI (x 10^ 3, 3, 3, 2, 3, 3, 3, 4, respectively) are shown below for 4-5 mice per group. Data shown are means ± SEM, representative of ≥ 2 experiments. Significance was defined by two-way ANOVA with the Holm-Šídák's multiple comparisons test, where *$p < 0.05$, **$p < 0.01$, and ***$p < 0.001$. (**D**) Naïve CD8 T cells from the spleens of CAR- or control-treated mice from Figure 1 were analyzed for levels of Foxo1 and CD127. Data are means ± SEM, from 2 experiments combined. (**E**) Gating strategy. (**F**) Representative CD19 staining for all 4 patient groups. g, Summarized results showing CD19+ cells as a percentage of total live PBMCs for each subject. Data shown are means ± SEM, where n = 19, 8, 20, and 19, respectively.

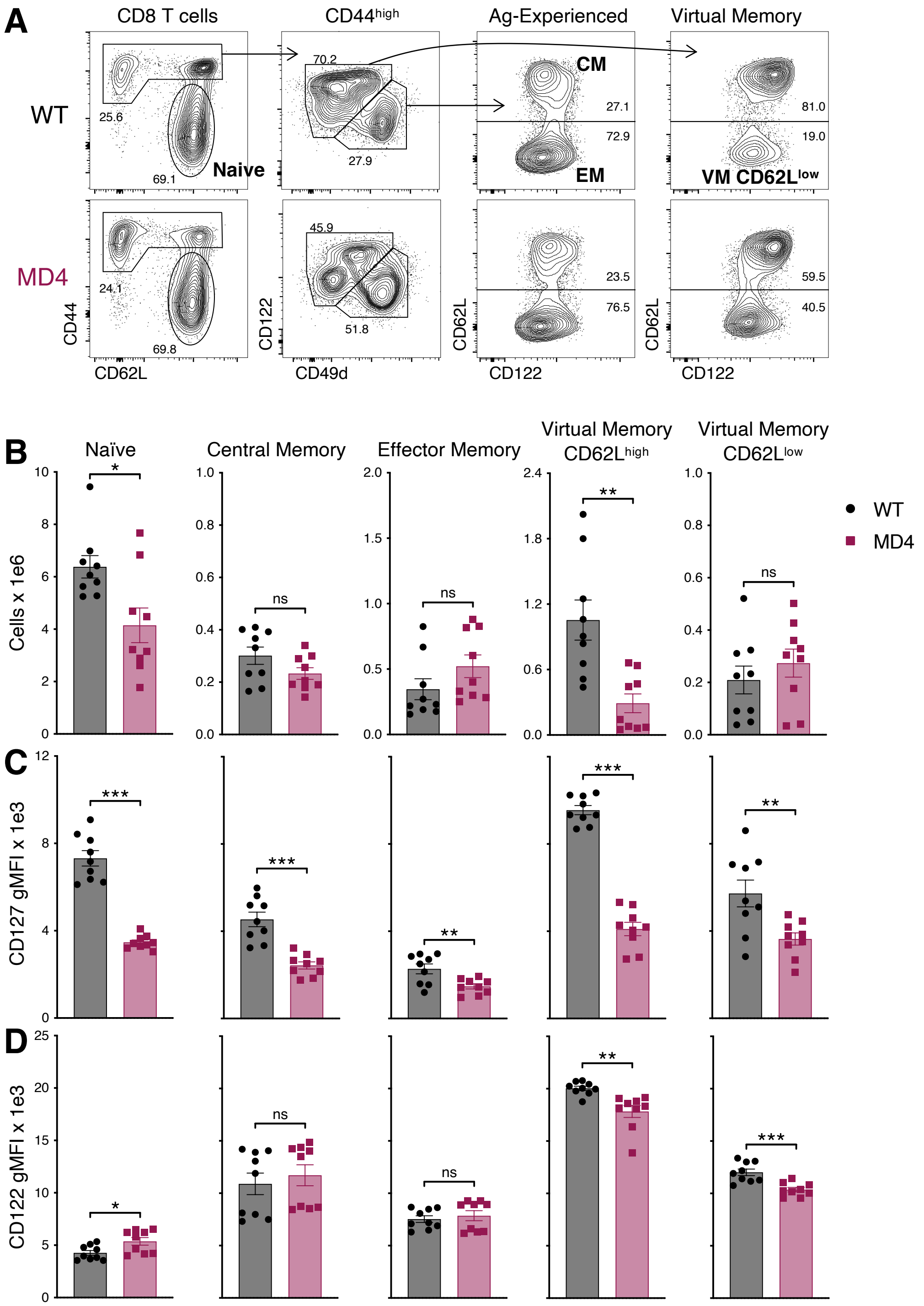

Figure S3. Naïve CD8 T cells from B cell-restricted hosts exhibit normal proliferative capacity, but defective survival. (**A**) Representative flow cytometry gating, pre-gated on live, CD8 T cells in unmanipulated WT or MD4 mice. Arrows indicate population hierarchies. (**B**) Total splenic cell numbers for the given CD8 T cell subsets. (**C**) gMFI of CD127, and d, gMFI of CD122 for the cell subsets. Data shown are means ± SEM, from 2 experiments combined.

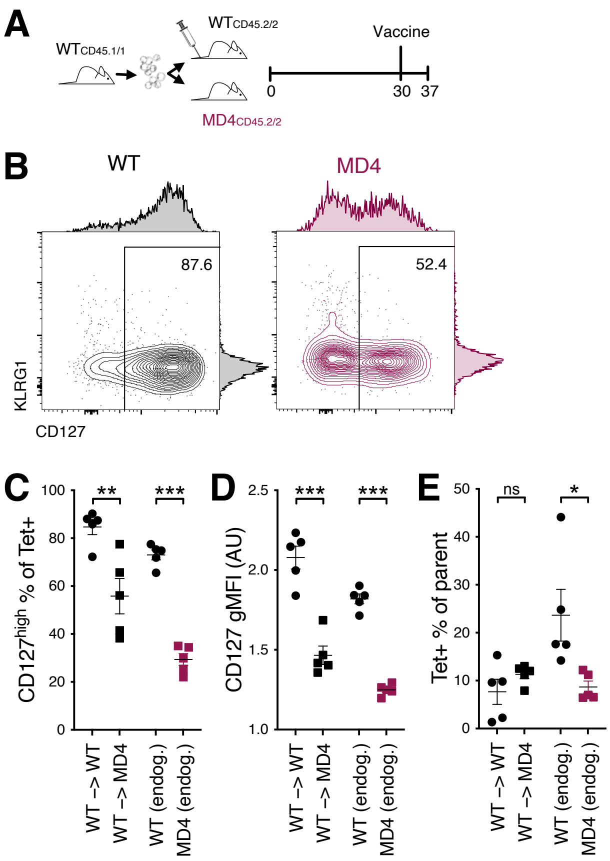

Figure S4. The B cell environment in which a naïve CD8 T cell develops has significant consequences on its response to vaccination. (**A-D**) Four million purified, congenically distinct polyclonal CD8 T cells from WT mice were transferred IV into WT or MD4 recipients. One month later, mice were vaccinated with the combined adjuvant protein subunit vaccine. After 7 days, spleens were analyzed by flow cytometry. (**A**) Experimental schematic. (**B**) Percentage of tetramer+ cells expressing high levels of CD127. (**C**) The gMFI of CD127 staining on tetramer+ CD127^{high} cells. (**D**) The gMFI of CD122 staining on tetramer+ cells. (**E**) The percentage of tetramer+ cells within CD45.1/1 transferred CD8 T cells (left columns) or CD45.2/2 endogenous CD8 T cells (right columns). Data shown are means ± SEM, n = 5 mice per group. Experiment using polyclonal, non-Tg CD8 T cells was performed once.

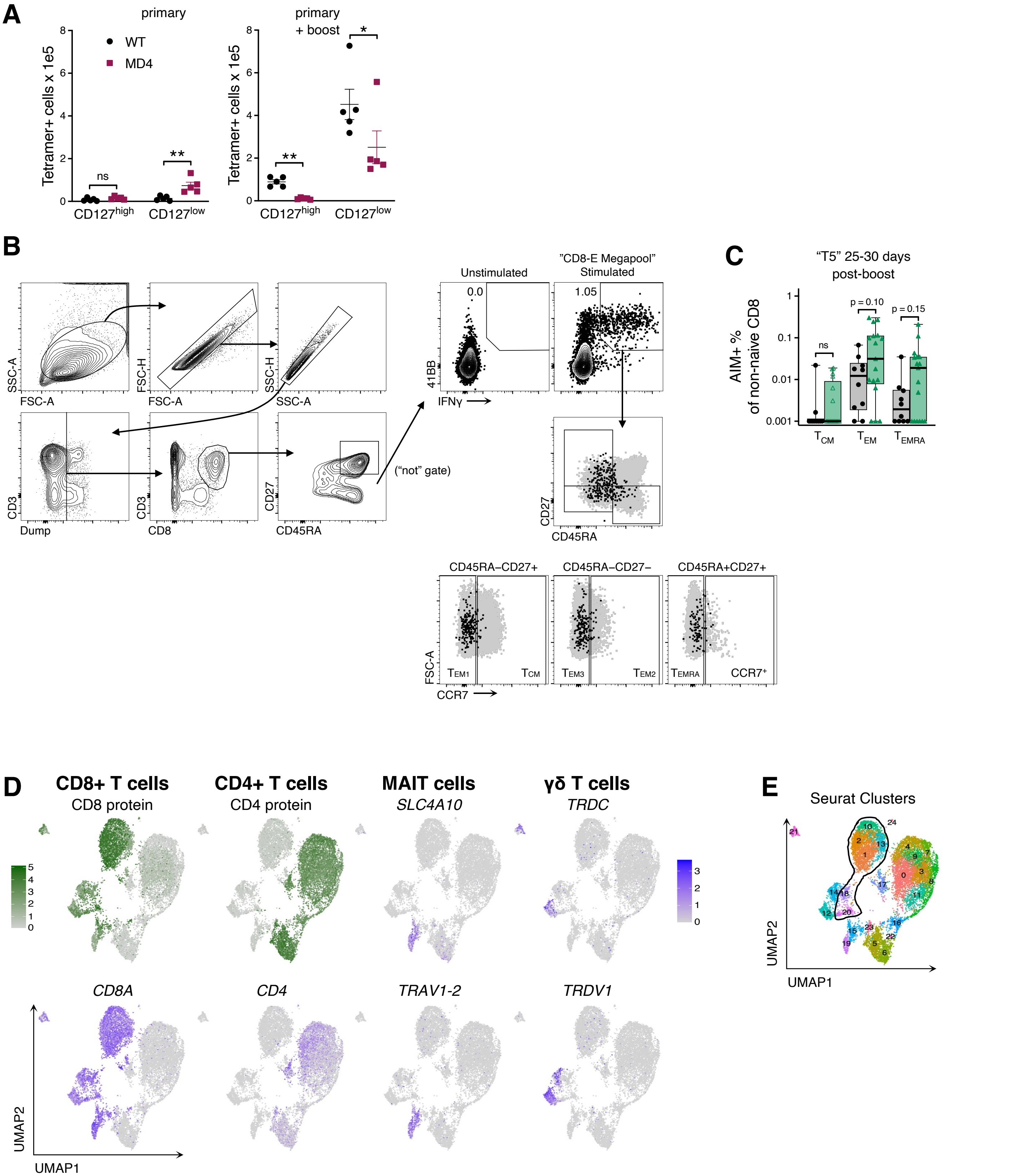

Figure S5. B cells limit effector CD8 T cell expansion following mRNA lipid nano-particle vaccination, preserving memory pool. (**A**) Mice were given either a primary mRNA LNP vaccination only (left) or a primary vaccine followed by a booster 30 days later (right). Spleens were assessed for the number of tetramer+ CD127^{high} cells and tetramer+ CD127^{low} cells. (**B**) Gating strategy for analysis of Ag-specific CD8 T cells using publicly available data from Bar-Or and colleagues. (**C**) AIM+ cells as a percent of non-naïve CD8 T cells at 25-30 days post vaccination boost. (**D**) UMAP visualizations of all cells sequenced split by CITE-seq antibody or gene, for the identification and exclusion of CD4 T cells, MAIT cells, and γδT cells. (**E**) UMAP visualization showing the clusters selected for downstream, conventional CD8 T cell analyses.

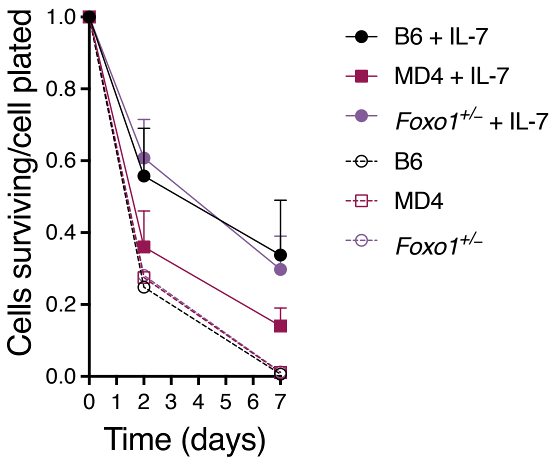

Figure S6. FOXO1 haploinsufficient CD8 T cells closely resemble those deprived of B cell help. Purified CD8 T cells from WT, *E8I-Cre Foxo1*$^{WT/FL}$, and MD4 mice were plated in vitro with or without 5 ng/ml of human IL-7. The number of live cells counted compared to the number of cells plated is displayed for days 0, 2, and 7. Data shown are combined from 2 separate experiments, where each n-value equals the average of all the replicates from one experiment.
